# Nano-flow cytometry unveils mitochondrial permeability transition process and multi-pathway cell death induction for cancer therapy

**DOI:** 10.1038/s41420-024-01947-y

**Published:** 2024-04-15

**Authors:** Liyun Su, Jingyi Xu, Cheng Lu, Kaimin Gao, Yunyun Hu, Chengfeng Xue, Xiaomei Yan

**Affiliations:** Department of Chemical Biology, MOE Key Laboratory of Spectrochemical Analysis & Instrumentation, Key Laboratory for Chemical Biology of Fujian Province, State Key Laboratory of Physical Chemistry of Solid Surfaces, Collaborative Innovation Center of Chemistry for Energy Materials, College of Chemistry and Chemical Engineering, Xiamen University, Xiamen, Fujian People’s Republic of China

**Keywords:** Apoptosis, Microfluidics

## Abstract

Mitochondrial permeability transition (mPT)-mediated mitochondrial dysfunction plays a pivotal role in various human diseases. However, the intricate details of its mechanisms and the sequence of events remain elusive, primarily due to the interference caused by Bax/Bak-induced mitochondrial outer membrane permeabilization (MOMP). To address these, we have developed a methodology that utilizes nano-flow cytometry (nFCM) to quantitatively analyze the opening of mitochondrial permeability transition pore (mPTP), dissipation of mitochondrial membrane potential ($\Delta$Ψ_m_), release of cytochrome c (Cyt c), and other molecular alternations of isolated mitochondria in response to mPT induction at the single-mitochondrion level. It was identified that betulinic acid (BetA) and antimycin A can directly induce mitochondrial dysfunction through mPT-mediated mechanisms, while cisplatin and staurosporine cannot. In addition, the nFCM analysis also revealed that BetA primarily induces mPTP opening through a reduction in Bcl-2 and Bcl-xL protein levels, along with an elevation in ROS content. Employing dose and time-dependent strategies of BetA, for the first time, we experimentally verified the sequential occurrence of mPTP opening and $\Delta$Ψ_m_ depolarization prior to the release of Cyt c during mPT-mediated mitochondrial dysfunction. Notably, our study uncovers a simultaneous release of cell-death-associated factors, including Cyt c, AIF, PNPT1, and mtDNA during mPT, implying the initiation of multiple cell death pathways. Intriguingly, BetA induces caspase-independent cell death, even in the absence of Bax/Bak, thereby overcoming drug resistance. The presented findings offer new insights into mPT-mediated mitochondrial dysfunction using nFCM, emphasizing the potential for targeting such dysfunction in innovative cancer therapies and interventions.

## Introduction

Mitochondrial permeability transition (mPT)-mediated mitochondrial dysfunction plays a critical role in the pathophysiology of various human diseases, including neurodegenerative disorders, ischemia-reperfusion injury, and cardiovascular diseases [1]. mPT involves a rapid increase in permeability of the inner mitochondrial membrane (IMM) through the opening of the mitochondrial permeability transition pore (mPTP) [2]. Overloading of Ca^2+^, excessive reactive oxygen species (ROS), and various endogenous regulators trigger the opening of mPTP [3]. This allows solutes below 1.5 kDa to enter the mitochondrial matrix, resulting in mitochondrial swelling, dissipation of the mitochondrial membrane potential ($\Delta$Ψ_m_), disruption of the outer mitochondrial membrane (OMM), and the release of intermembrane space (IMS) proteins, ultimately leading to cell death [4, 5]. While the occurrence and consequences of mPT are well-recognized, Bax/Bak-induced mitochondrial outer membrane permeabilization (MOMP) can yield similar outcomes [3, 6, 7]. In addition, the physical interaction of Bax/Bak with the mPTP constituents on the OMM can trigger mPT [8, 9]. Due to a limited understanding of mPTP structural and its intricate interplay, the molecular complexities of mPT, including the sequence of events related to $\Delta$Ψ_m_ decline and the release of Cyt c, as well as the factors contributing to cell death released during this process, remain elusive [10–19]. Therefore, mPT-mediated mitochondrial dysfunction and cell death have significant implications in cancer treatment beyond pathological diseases, necessitating further investigation [4, 20].

Isolated mitochondria experiments offer a direct means of evaluating the effect of external stimuli while circumventing the interference of intrinsic mPTP modulators such as ADP, Mg^2+^, H^+^, and cytoplasmic Bax [3, 21]. Monitoring mPTP opening typically involves the use of the swelling technique through 540 nm absorption measurement and the Ca^2+^-retention capacity method employing a Ca^2+^-sensitive fluorescence probe [22, 23]. Nevertheless, these ensemble-averaged methods are unable to unveil mitochondrial heterogeneity or sub-populations with different mPT tendencies [24, 25]. While electrophysiology measures channel current in individual mitoplasts (mitochondria with partially removed OMM), it only provides information on the status of mPTP opening [26]. In order to gain a comprehensive understanding of the complete process of mPT-mediated mitochondrial dysfunction, it is imperative to utilize multiparameter measurements for precise assessment of mPTP opening and the related molecular changes. While flow cytometry is well-suited for single-cell analysis, its application to individual mitochondria is constrained by its limited sensitivity [27]. Consequently, advanced technologies are required to measure various parameters and molecular events associated with mPT-mediated mitochondrial dysfunction, including cell death factors and mPTP modulators.

By integrating light scattering with strategies for single-molecule fluorescence detection in a sheathed flow, our laboratory has pioneered the development of nano-flow cytometry (nFCM), alternatively known as a high-sensitivity flow cytometer (HSFCM). This innovation facilitates the detection of nanoscale biological particles and organelles [28–31]. Expanding on nFCM, we have developed sensitive approaches for measuring $\Delta$Ψ_m_, Bcl-2, and Bax copy numbers, and mitochondrial fusion efficiency at the single-mitochondrion level [28, 32–34]. In this study, we applied nFCM to establish a quantitative method for detecting mPTP opening, $\Delta$Ψ_m_ loss, Cyt c release, and porin reduction in single mitochondria upon mPT induction. We identified compounds that directly induce mPTP opening and employed dose and time-dependent strategies to reveal the sequence of events involving $\Delta$Ψ_m_ loss and Cyt c release. Our study unveiled simultaneous release of cell death factors, including Cyt c, AIF, PNPT1, and mtDNA during mPT, implying the initiation of multiple cell death pathways. Significantly, BetA induces caspase-independent cell death even in the absence of Bax/Bak, thus overcoming drug resistance. These findings highlight mPTP as a promising approach in cancer therapy and shed light on its regulatory mechanisms, offering new prospects for therapeutic interventions.

## Results

### Development of the nFCM-based approach for determining mPT and associated changes in individual mitochondria

**Fig. 1:**
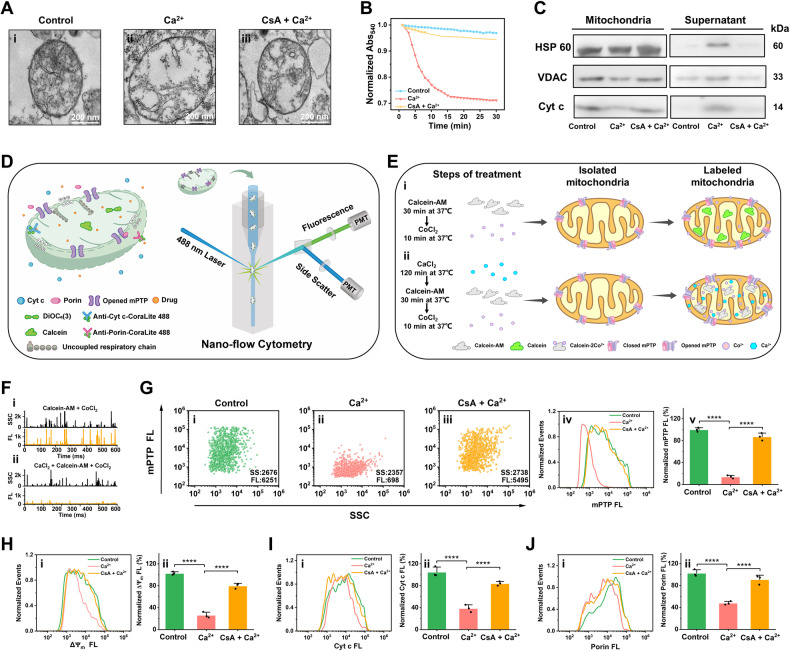
Development of the nFCM-based approach for assessing mPTP opening and related changes of individual mitochondria. **A** Representative transmission electron microscopic images of mitochondria isolated from HeLa cells: (i) Control mitochondria without treatment. (ii) Mitochondria treated with 400 μM CaCl_2_ for 2 h. (iii) Mitochondria pre-incubated with 10 μM CsA for 30 min before CaCl_2_ treatment (Scale bars, 200 nm). CsA, an mPTP inhibitor, binds to mitochondrial matrix cyclophilin D, preventing pore opening. **B** Mitochondrial swelling measured by absorbance at 540 nm upon treatment with 400 μM CaCl_2_. Isolated mitochondria from HeLa cells were resuspended in swelling buffer at a concentration of 1 mg/mL for 30 min. **C** Western blot analysis of mitochondrial proteins (Cyt c, VDAC, and HSP 60) in both the mitochondria and supernatant fractions under different treatment conditions. **D** Schematic representation illustrating the use of nFCM to monitor mPTP opening and changes in $\Delta$Ψ_m_, Cyt c in the mitochondrial intermembrane space, and porin in the mitochondrial outer membrane at the single-mitochondrion level. **E** Schematic design for the analysis of mPTP opening by nFCM. (i) Mitochondria are initially loaded with the acetoxymethyl ester of calcein dye, calcein AM, which passively diffuses into bilayer membrane and accumulates in mitochondrial matrix. Esterases cleave the acetoxymethyl esters, releasing the highly polar fluorescent dye calcein. Calcein, unable to penetrate mitochondrial membranes, emits a green fluorescence. (ii) Upon Ca^2+^-induced opening of the mPTP, Co^2+^, initially impermeable to the IMM, flows into the matrix and quenches the fluorescence of calcein. **F** Representative side scatter (SSC) and green fluorescence (FL) burst traces obtained from 60 s of data acquisition for calcein-AM stained mitochondria (i), and mitochondria treated with 400 μM CaCl_2_ for 2 h and then stained with calcein-AM (ii), both finally supplemented with CoCl_2_. **G** Bivariate dot-plots of mPTP fluorescence versus the side scatter of isolated mitochondria without treatment (i), treated with 400 μM CaCl_2_ for 2 h (ii), and pre-incubated with 10 μM CsA for 30 min before CaCl_2_ treatment (iii). Alongside histograms depicting the mPTP fluorescence distribution (iv) and the normalized median fluorescence intensity (v) for isolated mitochondria with different treatments. **H**–**J** Distribution histograms (i) and the normalized median fluorescence intensity (ii) of $\Delta$Ψ_m_ (**H**), Cyt c (**I**), and porin (**J**) for isolated mitochondria with different treatments. Error bars represent the mean ± standard error from three independent experiments, with statistical significance determined by paired t-test analysis. ****p < 0.0001 and n. s., non-significant.

Mitochondria were isolated from HeLa cells, and elevated levels of Ca^2+^ were used as a model system to induce mPT [35]. Seahorse Analyzer measurements of basal and ADP-stimulated oxygen consumption rates revealed the preserved functionality of oxidative phosphorylation (OXPHOS) in isolated mitochondria from HeLa cell. The introduction of 4 mM ADP resulted in a substantial enhancement of the oxygen consumption rate (Supplementary Fig. S1). To validate mPT, we employed various methods before developing the nFCM-based approach for measurements at the individual-mitochondrion level. These methods included electron microscopy for assessing mitochondrial morphology, spectrophotometry to monitor Ca^2+^-induced mitochondrial swelling, western blotting to detect Cyt c release and porin loss upon OMM rupture, and the inhibition of mPTP opening using cyclosporin (CsA) (Fig. 1A–C). It is worth noting that drug molecules within the cytosol of cells undergo concentration several hundred times higher than the extracellular concentration, spanning from nanomolar to micromolar levels. Isolated mitochondria exhibit enhanced tolerance to CsA concentrations, such as 10 μM, surpassing cellular levels (Supplementary Fig. S1) [14–18, 34]. Subsequently, mPT and related changes were monitored at the single-mitochondrion level using nFCM (Fig. 1D). Isolated mitochondria were stained with membrane-permeable dye calcein-AM and CoCl_2_, 3,3 dihexyloxacarbocyanine iodide (DiOC_6_(3)), and antibodies against Cyt c or porin to assess mPTP status, $\Delta$Ψ_m_ changes, and OMM integrity based on Cyt c and porin content, respectively.

When the mPTP is closed, calcein-AM is cleaved by esterase after crossing the mitochondrial membrane, leading to the formation of membrane-impermeable fluorescent calcein trapped within the mitochondrial matrix (Fig. 1E (i)). The opening of mPTP induced by Ca^2+^ results in Co^2+^ entry and a reduction in calcein fluorescence (Fig. 1E (ii)). Representative side scatter (SSC) and fluorescence (FL) burst traces obtained for the 5 μM calcein-AM stained mitochondria without and with Ca^2+^ treatment are displayed in Fig. 1F. The bivariate dot-plot of calcein fluorescence versus SSC displays strong green fluorescence for isolated mitochondria incubated with calcein-AM and CoCl_2_ (control sample, Fig. 1G (i)). After 2 h of treatment with 400 μM Ca^2+^ at 37 °C, a 90% decrease in calcein fluorescence was observed due to Co^2+^ entry (Fig. 1G (ii)). In contrast, mitochondria pre-treated with 10 μM CsA for 30 min at 37 °C exhibited nearly unchanged fluorescence after Ca^2+^ treatment (Fig. 1G (iii)). A clear trend is evident in the calcein fluorescence distribution histograms (Fig. 1G (iv)), and the normalized median fluorescence bar graph demonstrates a significant quenching of calcein fluorescence by Co^2+^ upon Ca^2+^-induced mPT, which is mitigated by CsA (Fig. 1G (v)).

Under normal conditions, $\Delta$Ψ_m_ ranges from 120 to 180 mV, with the inner mitochondrial side being electronegative [36]. Histograms of DiOC_6_(3) fluorescence (Fig. 1H (i)), along with the normalized median fluorescence bar graphs (Fig. 1H (ii)), demonstrate a decrease in $\Delta$Ψ_m_ due to Ca^2+^-induced mPT, which was effectively preserved by CsA inhibition. To assess Cyt c content in the IMS using immunofluorescence staining, we conducted mitochondria fixation and permeabilization. Mitochondria were incubated with anti-Cyt c monoclonal antibody (mAb) at a concentration of 20 μg/mL, followed by labeling with CoraLite 488-Conjugated AffiniPure IgG. Histograms of Cyt c fluorescence were generated for the control, Ca^2+^ induction, and CsA inhibition (Fig. 1I (i)). The accompanying normalized median fluorescence bar graphs reveal that Ca^2+^-induced mPT resulted in approximately 60% release of Cyt c from IMS upon OMM rupture, a process effectively prevented by CsA (Fig. 1I (ii)). Similarly, a substantial reduction in OMM protein porin was observed in mitochondria following Ca^2+^-induced mPT, with CsA confirming its ability to prevent mPTP opening (Fig. 1J).

Clearly, nFCM offers sensitive detection of mPT, $\Delta$Ψ_m_ depolarization, Cyt c release, and porin reduction following Ca^2+^ treatment, along with the effective inhibition of mPTP opening by CsA at the single-mitochondrion level. Importantly, in comparison to conventional spectrophotometric methods, the nFCM assay requires approximately 20-fold less sample quantity, which is particularly advantageous when assessing rare mitochondrial samples from patients with mitochondrial diseases.

### Identification of anticancer compounds that directly induce mitochondrial dysfunction through mPT

**Fig. 2:**
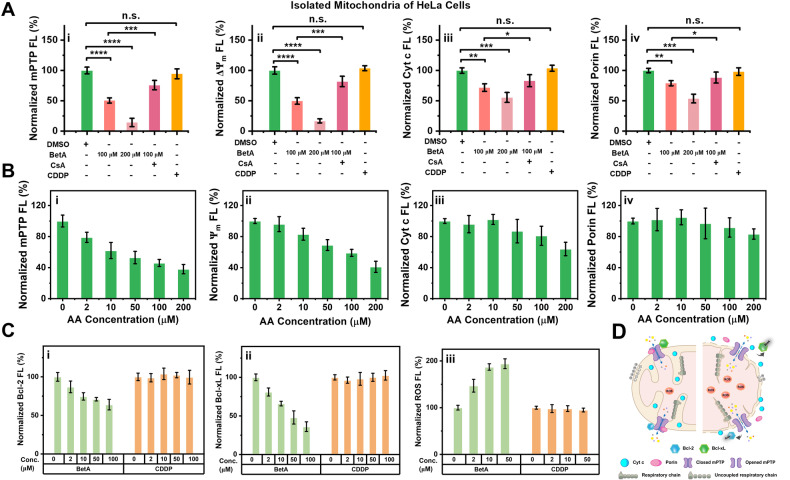
Identification of direct induction of mPT-mediated dysfunction in isolated mitochondria from HeLa cells by anticancer drugs using nFCM. **A** Normalized median fluorescence intensity for mPTP (i), $\Delta$Ψ_m_ (ii), Cyt c (iii), and porin (iv) in isolated mitochondria obtained through nFCM. Isolated mitochondria from HeLa cells were treated with 0.5% DMSO (control), 100 μM BetA, 200 μM BetA, 100 μM BetA after pre-incubation with 10 μM CsA for 30 min, and 200 μM CDDP for 2 h. **B** Normalized median fluorescence of mPTP (i), $\Delta$Ψ_m_ (ii), Cyt c (iii), and porin (iv) in isolated mitochondria stimulated with different concentrations of antimycin A (AA) for 2 h. **C** Levels of anti-apoptotic proteins Bcl-2 (i) and Bcl-xL (ii) in individual mitochondria following 2 h of treatment with 0, 2, 10, 50, or 100 μM BetA, and the content of ROS (iii) in individual mitochondria after being treated with 0, 2, 10, or 50 μM BetA. **D** Schematic diagram describing the mechanism of BetA-induced mPTP opening. BetA reduces the abundance of anti-apoptotic proteins Bcl-xL and Bcl-2 on the OMM and disrupts the mitochondrial respiratory chain by generating excessive ROS, ultimately leading to mPTP opening. Error bars represent the mean ± standard error from three independent experiments, and statistical significance was determined using paired t-test analysis. ****P < 0.0001, ***P < 0.001, **P < 0.01, *P < 0.05, and n. s., non-significant.

Different classes of anticancer compounds activate distinct signaling pathways to induce cell death. We employed the newly-developed approach for monitoring mPT and related mitochondrial changes to investigate whether anticancer compounds can directly trigger mPT-mediated mitochondrial dysfunction. Anticancer compounds, including betulinic acid (BetA), cisplatin (CDDP), antimycin A (AA), and staurosporine (STS), were incubated with mitochondria isolated from HeLa cells for 2 h at 37 °C. To validate mPTP opening, CsA inhibition experiments were conducted. Bivariate dot-plots of green fluorescence versus SSC provided a comprehensive examination of mPTP status, $\Delta$Ψ_m_ levels, Cyt c release, and porin content in individual mitochondria (Supplementary Fig. S2). As shown in the normalized median fluorescence bar graphs, treatment with 100 μM and 200 μM BetA significantly induced mPTP opening, decreased $\Delta$Ψ_m_, released Cyt c, and reduced porin levels (Fig. 2A). Pre-incubation with 10 μM CsA effectively prevented mPTP induced by 100 μM BetA (Fig. 2A). However, 200 μM CDDP did not lead to significant changes. Similar results were observed in mitochondria isolated from the MDA-MB-231 cell line (Supplementary Fig. S3). We also investigated the effects of AA and STS on HeLa cells mitochondria (Fig. 2B and Supplementary Fig. S4). BetA and AA showed a dose-dependent decrease in mPTP opening, $\Delta$Ψ_m_, Cyt c, and porin fluorescence, whereas CDDP and STS did not directly induce mPT and mitochondrial dysfunction.

The regulation of mPT-mediated mitochondrial dysfunction involves the Bcl-2 family proteins and ROS [1, 3]. These features were also measured during BetA-induced mPTP opening. nFCM provided the necessary sensitivity to quantitatively measure low-abundance anti-apoptotic Bcl-2 and Bcl-xL proteins in individual mitochondria (Supplementary Fig. S5). Treatment with BetA for 2 h resulted in a concentration-dependent decrease in Bcl-2 and Bcl-xL levels in mitochondria, with a more significant reduction observed for Bcl-xL, while CDDP had no apparent effect (Fig. 2C (i and ii)). The findings suggest that BetA may directly induce structural alterations or cleavage of the Bcl-2 and Bcl-xL protein on the OMM, even at a low concentration of 2 µM [37]. Mitochondrial ROS production was assessed using MitoSOX Red [38], as shown in Fig. 2C (iii) and Supplementary Fig. S6, demonstrating a concentration-dependent increase in ROS production with 2 h of BetA treatment, but almost no enhancement with CDDP. Further investigation revealed that BetA enhanced ROS production in mitochondria within 5 min, with a dose-dependent effect (Supplementary Fig. S7A, B). The Seahorse Analyzer revealed that BetA at concentrations of 10 µM and 50 µM effectively diminish both basal and ADP-stimulated oxygen consumption rates, as illustrated in Supplementary Fig. S8. This observation implies that the generation of ROS is a consequence of respiratory chain disruption. The concurrent reduction in Bcl-2 and Bcl-xL levels further contributes to the initiation of mitochondrial permeability transition pore (mPTP) opening, as depicted in Fig. 2D.

### Investigating the detailed process of mPT-mediated mitochondrial dysfunction for mechanistic study

**Fig. 3:**
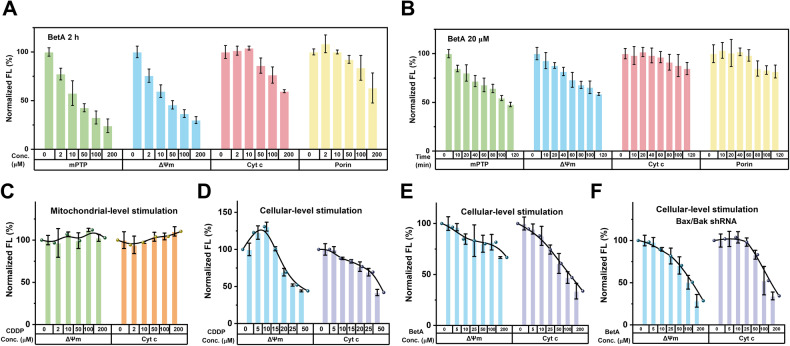
Investigating the detailed process of mPT-mediated mitochondrial dysfunction using nFCM. **A** Isolated mitochondria were exposed to varying concentrations of BetA—0, 2, 10, 50, 100, or 200 μM BetA for 2 h. Subsequently, the fluorescence signals of mPTP, $\Delta$Ψ_m_, Cyt c, and porin in single mitochondria were detected using nFCM. **B** Isolated mitochondria were treated with 20 μM BetA for different durations—0, 10, 20, 40, 60, 80, 100, or 120 min. Following treatment, the fluorescence of mPTP, $\Delta$Ψ_m_, Cyt c, and porin in single mitochondria were assessed by nFCM. **C** Isolated mitochondria were exposed to various concentrations of CDDP for 2 h, and the levels of $\Delta$Ψ_m_ and Cyt c were analyzed at the single-mitochondrion level using nFCM. **D**, **E** HeLa cells were treated with different concentrations of CDDP (**B**) or BetA (**C**) for 24 h. Mitochondria were then isolated, and the levels of $\Delta$Ψ_m_ and Cyt c were analyzed at the single-mitochondrion level using nFCM. **F** Bax/Bak double-knockdown HeLa cells were exposed to varying concentrations of BetA for 24 h. Subsequently, mitochondria were isolated, and the levels of $\Delta$Ψ_m_ and Cyt c were analyzed using nFCM.

To comprehensively understand the mechanisms of mPT-mediated mitochondrial dysfunction, we conducted an in-depth analysis to investigate the dose and time dependencies of the mitochondrial response to BetA. Figure 3A shows the normalized fluorescence levels of mPTP, $\Delta$Ψ_m_, Cyt c, and porin for isolated mitochondria following 2 h of treatment with varying concentrations of BetA. Even at 2 μM BetA, approximately 20% mPTP opening and $\Delta$Ψ_m_ dissipation were observed, which increased to nearly 60% at 50 μM BetA, accompanied by Cyt c release and porin reduction. Higher BetA concentrations (100 μM and 200 μM) intensified these effects. Although all factors exhibited a dose-dependent response, Cyt c and porin displayed delayed changes concerning BetA concentration. Subsequently, we exposed the mitochondrial suspension to 20 μM BetA for varying durations. Figure 3B shows that mPTP opening and $\Delta$Ψ_m_ dissipation occurred as early as 10 min, while Cyt c release and porin reduction were observed between 60 to 80 min. Notably, mPTP opening slightly exceeded $\Delta$Ψ_m_ dissipation (more than 50% versus less than 50% at 120 min). Comparing the time-dependent trends of mPTP, $\Delta$Ψ_m_, Cyt c, and porin fluorescence, we conclude that a more substantial mPTP opening and $\Delta$Ψ_m_ reduction are required to trigger OMM rupture, Cyt c release, and porin reduction. Leveraging the rapid and highly sensitive nFCM analysis, for the first time, we experimentally demonstrated that mPTP opening and $\Delta$Ψ_m_ depolarization precede Cyt c release and porin reduction in mPT-mediated mitochondrial dysfunction at the single-mitochondrion level.

Previous reports have suggested that in HeLa cells without mPTP opening, Cyt c release precedes $\Delta$Ψ_m_ loss [6, 12, 39]. However, our data indicate that BetA-induced mPTP opening results in an earlier loss of $\Delta$Ψ_m_ than Cyt c release (Fig. 3A, B). To address this discrepancy, we conducted BetA and CDDP treatments at both mitochondrial and cellular levels. In Fig. 2, CDDP did not induce mPT-mediated mitochondrial dysfunction, and mitochondrial treatment with varying CDDP concentrations for 2 h showed no notable changes in $\Delta$Ψ_m_ or Cyt c levels (Fig. 3C). In contrast, isolated mitochondria from HeLa cells treated with different CDDP concentrations for 24 h exhibited a dose-dependent response in $\Delta$Ψ_m_ and Cyt c levels. While the extent of Cyt c release increased with the concentration of CDDP and could be observed at concentrations as low as 5 μM CDDP, $\Delta$Ψ_m_ initially increased with the CDDP concentration and decreased at around 15 μM (Fig. 3D, Supplementary Fig. S9A, B). These data suggest that $\Delta$Ψ_m_ dissipation lags behind the release of Cyt c during cell death induced by CDDP. A similar phenomenon was observed for STS, which did not induce mPT-mediated mitochondrial dysfunction at the mitochondrial level (Supplementary Fig. S4), and Cyt c release preceded $\Delta$Ψ_m_ decrease during cellular treatment (Supplementary Fig. S10).

In an effort to understand the differences in $\Delta$Ψ_m_ dissipation and Cyt c release observed when CDDP and STS were used to stimulate either at the mitochondrial or cellular level, we conducted cellular treatment with BetA before isolating the mitochondria for comparison. A clear and dose-dependent decrease in both $\Delta$Ψ_m_ and Cyt c fluorescence was observed in individual mitochondria when HeLa cells were exposed to varying concentrations of BetA for 24 h (Fig. 3E and Supplementary Fig. S11). Interestingly, the steeper decrease in Cyt c levels compared to $\Delta$Ψ_m_ contradicted the mitochondrial-level observation that BetA induced $\Delta$Ψ_m_ depolarization before Cyt c release (Fig. 3A, B). The release of Cyt c into the cytoplasm occurs following mPTP initiation or Bax/Bak oligomerization at the cellular level [6]. To minimize Bax and Bak interference, HeLa cells were genetically modified through stable transfection of short hairpin RNAs (shRNAs) specifically targeting Bax and/or Bak, generating three clones (Bax shRNA, Bak shRNA, and Bax/Bak shRNA). Immunoblotting analysis confirmed the negligible expression of Bax and Bak in the transfected HeLa cells (Supplementary Fig. S12). Then, the loss of $\Delta$Ψ_m_ and release of Cyt c were examined in HeLa cells with Bax/Bak double-knockdown using varying concentrations of BetA for 24 h (Fig. 3F and Supplementary Fig. S13). At low BetA doses, rapid $\Delta$Ψ_m_ dissipation was observed, while significant Cyt c release was detected only at around 25 μM BetA. These findings indicate that even in HeLa cells with Bax/Bak double-knockdown, Cyt c release can occur in response to BetA treatment, and the kinetics closely resemble those observed in isolated mitochondria treated with BetA (Fig. 3A). Specifically, $\Delta$Ψ_m_ dissipation precedes the release of Cyt c.

### mPT-mediated mitochondrial dysfunction leads to cell death through multiple pathways

Mitochondria serve as the executioners of cell demise, housing factors linked to cell death, such as AIF, PNPT1, and mtDNA, which are released into the cytoplasm during cell death [7, 40]. Using nFCM to detect changes in AIF, PNPT1, and mtDNA content in individual mitochondria allows for the investigation of their release in mPT-mediated mitochondrial dysfunction. To assess nFCM sensitivity in detecting AIF and PNPT1 in individual mitochondria, mitochondria isolated from HeLa cells were incubated with anti-PNPT1 polyclonal antibody or anti-AIF mAb at a concentration of 20 μg/mL, followed by labeling with CoraLite 488-Conjugated AffiniPure IgG. Meanwhile, a membrane-permeable nucleic acid dye, SYTO 62, was used to label mtDNA [28, 32]. Representative side scatter and green fluorescence burst traces, bivariate dot-plots of green fluorescence against SSC, and fluorescence distribution histograms of AIF and PNPT1 confirmed the ample sensitivity of nFCM (Supplementary Fig. S14A, B).

**Fig. 4:**
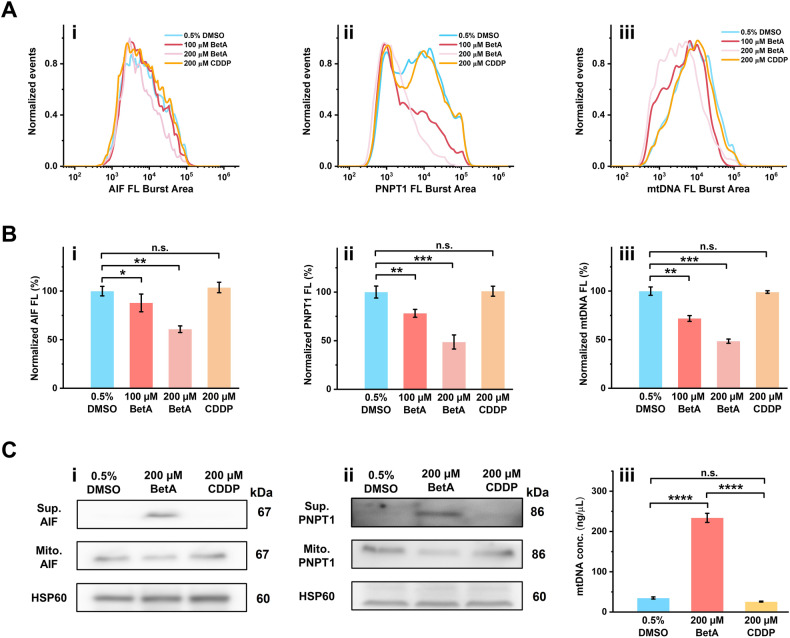
mPT-mediated mitochondrial dysfunction can lead to cell death through multiple pathways. **A**, **B** Fluorescence burst area distribution histogram (**A**) and normalized fluorescence burst area (**B**) of AIF protein (i), PNPT1 (ii), and mtDNA content (iii) in isolated mitochondria through single-mitochondrial analysis by nFCM. Isolated mitochondria were treated with 100 μM, 200 μM BetA, and 200 μM CDDP for 2 h, respectively. **C** Western blot measurement of AIF (i) and PNPT1 (ii) content in mitochondria and supernatant after stimulation with 200 μΜ BetA or CDDP. mtDNA concentration in supernatant was quantified in the unit of ng/μL using 1 μL aliquots of the sample via a SpectraMax QuickDrop Micro-Volume Spectrophotometer (iii). Error bars indicate the mean ± standard error of three independent experiments, and statistical significance was determined using paired t-test analysis. ****P < 0.0001, ***P < 0.001, **P < 0.01, *P < 0.05, and n. s., non-significant.

Subsequently, isolated mitochondria were treated with 0.5% DMSO, 100 μM BetA, 200 μM BetA, or 200 μM CDDP for 2 h. Following immunofluorescent staining or mtDNA labeling, mitochondrial samples were analyzed by nFCM. The fluorescence distribution histograms of AIF, PNPT1, and mtDNA displayed a leftward shift following BetA treatment compared to 0.5% DMSO, with a more pronounced shift at 200 μM BetA, while CDDP treatment had no discernable effect (Fig. 4A (i–iii)). The normalized median fluorescence bar graph clearly illustrated that 100 μM and 200 μM BetA, instead of 200 μM CDDP, significantly reduced AIF, PNPT1, and mtDNA levels within mitochondria, indicating their release (Fig. 4B (i–iii)). These findings were validated using immunoblotting and nucleic acids quantification using a quick drop spectrophotometer (Fig. 4C (i–iii)). In conclusion, BetA-induced mPTP opening leads to the comprehensive release of various cell-death-associated factors, initiating cascades that facilitate cell death induction, highlighting its potential as an effective anticancer therapy strategy.

### Promising chemotherapeutic potential of BetA for cancer treatment

**Fig. 5:**
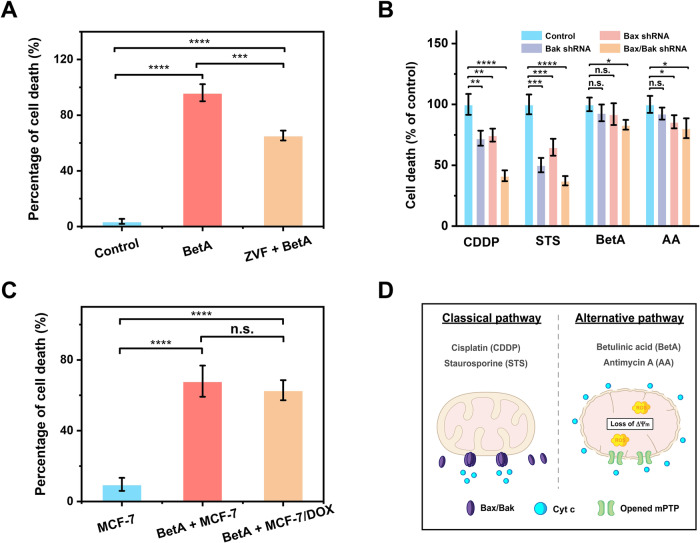
Powerful chemotherapeutic potential of BetA for cancer cell treatment. **A** HeLa cells were pre-treated with 100 μM Z-VAD(OMe)-FMK (ZVF) for 12 h before the addition of BetA. After 24 h of treatment with 100 μM of BetA, cell death was quantified using Annexin V/PI staining, followed by analysis on a BD FACSAria flow cytometer. **B** HeLa cells infected with lentivirus carrying a scrambled shRNA (control) or shRNAs targeting Bak (Bak shRNA), Bax (Bax shRNA), or both (Bax/Bak shRNA) were exposed to 100 μM CDDP, 500 nM STS, 100 μM BetA, or 100 μM AA for 24 h. Cell death was assessed using Annexin V/PI staining, followed by analysis on a BD FACSAria flow cytometer. **C** Cell death in MCF-7 and MCF-7 DOX cells was measured by flow cytometric analysis after treatment with 100 μM BetA for 24 h, using Annexin V/PI staining. **D** Schematic diagram illustrating cell death induction by BetA and AA, involving a Bax/Bak-independent and mPT-mediated mitochondrial dysfunction. Conversely, cell death triggered by CDDP and STS is reliant on Bax/Bak oligomerization. Error bars represent the mean ± standard error of three independent experiments, and statistical significance was determined using paired t-test analysis. ****P < 0.0001, ***P < 0.001, **P < 0.01, *P < 0.05, and n. s., non-significant.

Subsequently, an investigation into BetA’s potential for inducing cell death and cancer treatment was conducted. HeLa cells were exposed to 100 μM BetA, with or without the pan-caspase inhibitor Z-VAD(OMe)-FMK, for 24 h. Cell death was assessed through Annexin V/PI staining, revealing that even in the presence of caspase inhibitors, BetA-induced cell death persisted, albeit at a reduced rate (60% vs. 90%) (Fig. 5A and Supplementary Fig. S15). This suggests that BetA can trigger both caspase-dependent and caspase-independent cell death pathways.

Tumors often exhibit defects in Bax and Bak genes, potentially leading to drug resistance against therapies relying on these factors for cell death [41, 42]. Therefore, strategies that can overcome the deficiency of Bax and Bak hold promise in cancer treatment. To investigate their role in cell death at the cellular level, HeLa cell clones (Bax shRNA, Bak shRNA, and Bax/Bak shRNA) were exposed to 100 μM CDDP, 500 nM STS, 100 μM BetA, and 100 μM AA for 24 h. Cell death rates were measured, with the control cell line set at 100%, against which the knockdown cell lines were compared. Results revealed distinctions, with CDDP and STS showing significantly lower cell death rates in the knockdown cell lines, particularly the Bax/Bak double-knockdown cells with an approximately 60% reduction (Fig. 5B and Supplementary Fig. S16A, B). However, BetA and AA had a smaller impact on Bax and Bak silencing compared to CDDP and STS, resulting in a mere 20% difference in cell death between the HeLa control cell line and Bax/Bak shRNA cells (Fig. 5B and Supplementary Fig. S17A, B). These findings suggest that Bax/Bak-modified HeLa cells are resistant to STS and CDDP-induced cell death but remain susceptible to BetA and AA-induced cell death due to their ability to activate cell death pathways independent of Bax and Bak.

Drug resistance in breast cancer patients, particularly to medications like doxorubicin (DOX), is a significant challenge linked to drug efflux pumps expelling chemotherapy agents using ATP from mitochondria [43]. Overcoming drug resistance is an imminent concern that demands attention [44]. In this study, both MCF-7 and MCF-7/DOX cells were exposed to 100 μM BetA for 24 h, and cell death was evaluated using flow cytometry. The outcomes indicate no notable discrepancy in cell death between the two cell lines (Fig. 5C and Supplementary Fig. S18). This may be attributed to BetA’s ability to disrupt the mitochondrial respiratory chain, hindering ATP production, and deactivating the drug efflux pump, potentially overcoming chemotherapy resistance [45]. These findings suggest that Bax/Bak-mediated cell death and mPT-mediate cell death occur as distinct processes, as depicted schematically in Fig. 5D. BetA and AA directly induce mitochondrial dysfunction, thereby overcoming the resistance acquired upstream of the mitochondria. In contrast, the pathway performed inconspicuously in CDDP and STS-induced cell death.

## Discussion

The mPTP is a pivotal regulator of mitochondria, and its prolonged opening can result in cell death [2, 14]. Simultaneously, MOMP, initiated by the cytoplasmic protein Bax translocating to the OMM and forming oligomers, either with itself or with Bak, contributes to the mitochondrial-mediated death pathway [46]. Given the similarities in the mitochondrial dysfunction caused by these two scenarios, understanding the mechanisms of mPT-mediated cell death, particularly the sequence of events and the release of cell-death-associated factors during this process, remains challenging [3, 6, 7, 12]. To address this challenge, using isolated mitochondria to exclude cytoplasmic proteins and prevent interference from Bax/Bak-induced MOMP, while eliminating endogenous regulatory factors, has proven effective for investigating mPT-mediated mitochondrial dysfunction [21]. In this report, we have developed a high-throughput method utilizing nFCM technology to quantitatively measure mPT-mediated mitochondrial dysfunction parameters at the individual mitochondrial level, including mPTP opening, $\Delta$Ψ_m_ depolarization, Cyt c release, and porin protein reduction.

Previous studies have shown that compounds such as BetA, AA, STS, and CDDP induce mPT-mediated mitochondrial dysfunction at the cellular level [47–50]. However, our nFCM analysis with isolated mitochondria has revealed that BetA and AA directly induce mitochondrial dysfunction through mPT, while STS and CDDP do not have the same effect, likely due to the involvement of Bax and Bak at the cellular level. Furthermore, our nFCM analysis revealed that BetA primarily induces mPTP opening by reducing Bcl-2 and Bcl-xL protein levels and increasing ROS content (Fig. 2C). Consequently, our method holds promise for screening anticancer drugs or modulators that directly induce mPT-mediated mitochondrial dysfunction. When combined with genetic manipulation techniques such as protein knockout, this approach can aid in unraveling the composition and constituents of the mPTP.

Theoretically, mPTP opening leads to a rapid decrease in $\Delta$Ψ_m_, occurring prior to the release of Cyt c [6, 39, 51]. Through systematic stimulation of isolated mitochondria and nFCM analysis, we have provided the first experimental evidence confirming that $\Delta$Ψ_m_ depolarization precedes Cyt c release during BetA-induced mPT-mediated mitochondrial dysfunction (Fig. 3A, B). This result has been validated by experiments involving the reduction of Bax and Bak protein expression in cells (Fig. 3F). Hence, quantitative nFCM analysis provides valuable insights into the fundamental mechanisms underlying mPTP opening and its subsequent outcomes, clarifying the chronological relationship between $\Delta$Ψ_m_ decrease and Cyt c release during cell death induced by different drugs. The choice between mPTP opening and Bax/Bak oligomerization hinges on the specific drug used to activate the mitochondrial pathway.

**Fig. 6:**
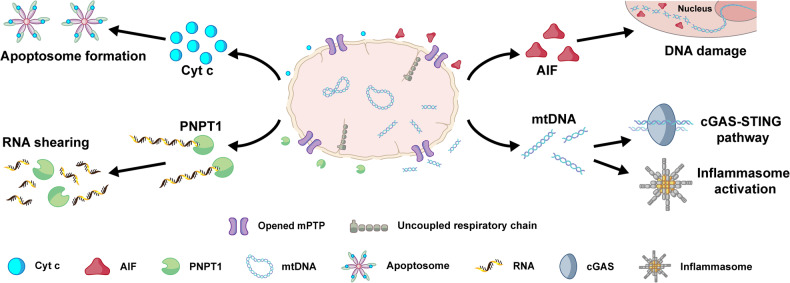
Schematic illustration of mitochondrial swelling and rupture following mPTP opening, leading to the release of multiple pro-death factors: Cyt c, AIF, PNPT1, and mtDNA. Upon mPTP opening, Cyt c is released into the cytoplasm, play a crucial role in triggering cell death pathways in a caspase-dependent manner through the formation of apoptosome. AIF translocates from the IMS to the cytoplasm and nucleus, inducing caspase-independent peripheral chromatin condensation and large-scale DNA fragmentation, contributing to cell death programs. In addition, the rupture of the outer membrane facilitates PNPT1 and mtDNA release into the cytoplasm. PNPT1, a 3’-to-5’ exoribonuclease, is released from the IMS, initiating the apoptotic decay of RNAs lacking 3’ structures and enhancing apoptosis by reducing the expression of unstable anti-apoptotic proteins. Meanwhile, the release of mtDNA induces inflammation and type I interferon responses via the cytoplasmic cGAS-STING DNA sensing pathway, thereby enhancing anti-tumor immune responses.

Our study has, for the first time, observed the simultaneous release of Cyt c, AIF, PNPT1, and mtDNA in the context of mPT-induced mitochondrial dysfunction (Figs. 2A (iii), 2B (iii) and 4). These factors initiate multiple pathways that promote cell death [7, 52–55], highlighting the potential of mPT-mediated mitochondrial dysfunction for eliminating cancer cells (Fig. 6). Moreover, defects in the cell death pathways upstream of mitochondria, such as reduced expression of pro-apoptotic Bcl-2 proteins (Bax and Bak) and caspases, as well as drug efflux pump expelling chemotherapy agents, contribute to the development of drug resistance in cells [56]. Our observations reveal that BetA, a drug that directly induces mPT-mediated mitochondrial dysfunction, possesses a remarkable ability to trigger caspase-independent cell death at the cellular level. It is also effective in killing cancer cells, even in the absence of Bax and Bak, thereby overcoming drug resistance in doxorubicin (DOX)-tolerant cell lines (Fig. 5C). In summary, by leveraging the advantages of nFCM to quantify mPTP opening and its subsequent consequences in individual mitochondria, we have unveiled the intricate mechanisms underlying mPT-mediated initiation of cell death. This understanding offers significant potential for innovative mitochondria-targeted anticancer strategies and overcoming cancer resistance.

## Materials and methods

Details of experimental protocols can be found in SI Appendix.

### Mitochondrial swelling assays

The investigation of mitochondrial swelling induced by high levels of Ca^2+^ was conducted using the method previously described by Schinzel et al. [57]. Isolated mitochondria (0.5 mg proteins) from all experimental groups were diluted into 200 μL of swelling buffer at 25 °C. Prior to exposure to CaCl_2_, the mitochondrial suspension in the treatment groups was supplemented with CsA (10 μM) and allowed to incubate for 30 min. The induction of swelling was achieved by the addition of 400 μM CaCl_2_, and the changes in light scattering at 540 nm were recorded using a microplate reader (SpectraMax iD5, Molecular Devices, San Jose, CA, USA) to monitor the decrease in light scattering at 540 nm for a duration of 30 min. Statistical analysis was performed using the variance in optical density (OD540) between the highest reading immediately subsequent to CaCl_2_ addition and the lowest reading obtained 30 min post addition.

### Nano-flow cytometry analysis

A laboratory-built nano-flow cytometer (nFCM) with a 488-nm laser excitation laser (10 mW) and three detection channels was used for the analysis of individual mitochondria [28]. The FL-1 channel (520/35 nm band-pass filter) and FL-2 channel (700/40 nm band-pass filter) were used to detect the green fluorescence (DiOC_6_, Calcein, and CoraLite 488) and red fluorescence (SYTO 62 and MitoSOX Red), respectively. Photon bursts were concurrently detected in the side scatter, green fluorescence (FL-1), and red fluorescence (FL-2) channels. The process of data acquisition and analysis remained consistent with those previously delineated. For each mitochondrial sample, a 60 s of data acquisition period was used. Additional information regarding the nFCM, including its parameter settings and data processing, can be gleaned from the elucidations put forth by Zhang et al. [28].

### Supplementary information

Supplementary material

WB Original file

## Data Availability

The data analyzed during this study are included in this published article and the supplemental data files. Additional supporting data are available from the corresponding authors upon reasonable request.

## References

[1] Cui Y, Pan M, Ma J, Song X, Cao W, Zhang P (2021). Recent progress in the use of mitochondrial membrane permeability transition pore in mitochondrial dysfunction-related disease therapies. Mol. Cell Biochem.

[2] Morciano G, Naumova N, Koprowski P, Valente S, Sardao VA, Potes Y (2021). The mitochondrial permeability transition pore: an evolving concept critical for cell life and death. Biol Rev Camb Philos Soc.

[3] Bonora M, Giorgi C, Pinton P (2022). Molecular mechanisms and consequences of mitochondrial permeability transition. Nat Rev Mol Cell Biol.

[4] Galluzzi L, Fulda S, Green DR, Martinou JC, Pinton P, Shao F (2018). Molecular mechanisms of cell death: recommendations of the Nomenclature Committee on Cell Death 2018. Cell Death Differ.

[5] Fricker M, Tolkovsky AM, Borutaite V, Coleman M, Brown GC (2018). Neuronal cell death. Physiol Rev.

[6] Fulda S, Galluzzi L, Kroemer G (2010). Targeting mitochondria for cancer therapy. Nat Rev Drug Discov.

[7] Bock FJ, Tait SWG (2020). Mitochondria as multifaceted regulators of cell death. Nat Rev Mol Cell Biol.

[8] Patel P, Mendoza A, Robichaux DJ, Wang MC, Wehrens XHT, Karch J (2021). Inhibition of the anti-apoptotic Bcl-2 family by BH3 mimetics sensitize the mitochondrial permeability transition pore through Bax and Bak. Front Cell Dev Biol.

[9] Karch J, Kwong JQ, Burr AR, Sargent MA, Elrod JW, Peixoto PM (2013). Bax and Bak function as the outer membrane component of the mitochondrial permeability pore in regulating necrotic cell death in mice. Elife.

[10] Flores-Romero H, Dadsena S, Garcia-Saez AJ (2023). Mitochondrial pores at the crossroad between cell death and inflammatory signaling. Mol Cell.

[11] Bernardi P, Carraro M, Lippe G (2022). The mitochondrial permeability transition: Recent progress and open questions. FEBS J.

[12] Wigdal SS, Kirkland RA, Franklin JL, Haak-Frendscho M (2002). Cytochrome c release precedes mitochondrial membrane potential loss in cerebellar granule neuron apoptosis: lack of mitochondrial swelling. J Neurochem.

[13] He J, Ford HC, Carroll J, Ding S, Fearnley IM, Walker JE (2017). Persistence of the mitochondrial permeability transition in the absence of subunit c of human ATP synthase. Proc Natl Acad Sci USA.

[14] Giorgio V, von Stockum S, Antoniel M, Fabbro A, Fogolari F, Forte M (2013). Dimers of mitochondrial ATP synthase form the permeability transition pore. Proc Natl Acad Sci USA.

[15] Galber C, Minervini G, Cannino G, Boldrin F, Petronilli V, Tosatto S (2021). The f subunit of human ATP synthase is essential for normal mitochondrial morphology and permeability transition. Cell Rep.

[16] Karch J, Bround MJ, Khalil H, Sargent MA, Latchman N, Terada N (2019). Inhibition of mitochondrial permeability transition by deletion of the ANT family and CypD. Sci Adv.

[17] Giorgio V, Burchell V, Schiavone M, Bassot C, Minervini G, Petronilli V (2017). Ca(2+) binding to F-ATP synthase beta subunit triggers the mitochondrial permeability transition. EMBO Rep.

[18] Antoniel M, Jones K, Antonucci S, Spolaore B, Fogolari F, Petronilli V (2018). The unique histidine in OSCP subunit of F-ATP synthase mediates inhibition of the permeability transition pore by acidic pH. EMBO Rep.

[19] Galber C, Fabbian S, Gatto C, Grandi M, Carissimi S, Acosta MJ (2023). The mitochondrial inhibitor IF1 binds to the ATP synthase OSCP subunit and protects cancer cells from apoptosis. Cell Death Dis.

[20] Waseem M, Wang BD (2023). Promising strategy of mPTP modulation in cancer therapy: An emerging progress and future insight. Int J Mol Sci.

[21] Bonora M, Morganti C, Morciano G, Giorgi C, Wieckowski MR, Pinton P (2016). Comprehensive analysis of mitochondrial permeability transition pore activity in living cells using fluorescence-imaging-based techniques. Nat Protoc.

[22] Bhosale G, Duchen MR (2019). Investigating the mitochondrial permeability transition pore in disease phenotypes and drug screening. Curr Opin Pharm.

[23] Marcu R, Neeley CK, Karamanlidis G, Hawkins BJ (2012). Multi-parameter measurement of the permeability transition pore opening in isolated mouse heart mitochondria. J Vis Exp.

[24] Biasutto L, Azzolini M, Szabo I, Zoratti M (2016). The mitochondrial permeability transition pore in AD 2016: an update. Biochim Biophys Acta Mol Cell Res.

[25] Lefebvre A, Ma D, Kessenbrock K, Lawson DA, Digman MA (2021). Automated segmentation and tracking of mitochondria in live-cell time-lapse images. Nat Methods.

[26] Neginskaya MA, Pavlov EV, Sheu SS (2021). Electrophysiological properties of the mitochondrial permeability transition pores: channel diversity and disease implication. Biochim Biophys Acta Bioenerg.

[27] Lian H, He SB, Chen CX, Yan XM (2019). Flow cytometric analysis of nanoscale biological particles and organelles. Annu Rev Anal Chem.

[28] Zhang S, Zhu S, Yang L, Zheng Y, Gao M, Wang S (2012). High-throughput multiparameter analysis of individual mitochondria. Anal Chem.

[29] Zhu SB, Ma L, Wang S, Chen CX, Zhang WQ, Yang LL (2014). Light-scattering detection below the level of single fluorescent molecules for high-resolution characterization of functional nanoparticles. ACS Nano.

[30] Ma L, Zhu SB, Tian Y, Zhang WQ, Wang S, Chen CX (2016). Label-free analysis of single viruses with a resolution comparable to that of electron microscopy and the throughput of flow cytometry. Angew Chem Int Ed.

[31] Tian Y, Ma L, Gong MF, Su GQ, Zhu SB, Zhang WQ (2018). Protein profiling and sizing of extracellular vesicles from colorectal cancer patients via flow cytometry. ACS Nano.

[32] Chen C, Zhang X, Zhang S, Zhu S, Xu J, Zheng Y (2015). Quantification of protein copy number in single mitochondria: the Bcl-2 family proteins. Biosens Bioelectron.

[33] Xu J, Su L, Han J, Gao K, Zhang M, Wang S (2021). Rapid and quantitative in vitro analysis of mitochondrial fusion and its interplay with apoptosis. Talanta.

[34] Zhang X, Zhang S, Zhu S, Chen S, Han J, Gao K (2014). Identification of mitochondria-targeting anticancer compounds by an in vitro strategy. Anal Chem.

[35] Hurst S, Hoek J, Sheu SS (2017). Mitochondrial calcium ion and regulation of the permeability transition pore. J Bioenerg Biomembr.

[36] Galluzzi L, Zamzami N, de La Motte Rouge T, Lemaire C, Brenner C, Kroemer G (2007). Methods for the assessment of mitochondrial membrane permeabilization in apoptosis. Apoptosis.

[37] Khan I, Guru SK, Rath SK, Chinthakindi PK, Singh B, Koul S (2016). A novel triazole derivative of betulinic acid induces extrinsic and intrinsic apoptosis in human leukemia HL-60 cells. Eur J Med Chem.

[38] Kauffman ME, Kauffman MK, Traore K, Zhu H, Trush MA, Jia Z (2016). Mitosox-based flow cytometry for detecting mitochondrial ROS. React Oxyg Species.

[39] Poppe M, Reimertz C, Dussmann H, Krohn AJ, Luetjens CM, Bockelmann D (2001). Dissipation of potassium and proton gradients inhibits mitochondrial hyperpolarization and cytochrome c release during neural apoptosis. J Neurosci.

[40] Bano D, Prehn JHM (2018). Apoptosis-inducing factor (AIF) in physiology and disease: the tale of a repented natural born killer. EBioMedicine.

[41] Kulbay M, Paimboeuf A, Ozdemir D, Bernier J (2022). Review of cancer cell resistance mechanisms to apoptosis and actual targeted therapies. J Cell Biochem.

[42] Blombery P, Thompson ER, Chen X, Huang DCS, Roberts AW, Anderson MA (2022). Clonal hematopoiesis, myeloid disorders and BAX-mutated myelopoiesis in patients receiving venetoclax for CLL. Blood.

[43] Halder J, Pradhan D, Kar B, Ghosh G, Rath G (2022). Nanotherapeutics approaches to overcome P-glycoprotein-mediated multi-drug resistance in cancer. Nanomedicine.

[44] Kanno Y, Chen CY, Lee HL, Chiou JF, Chen YJ (2021). Molecular mechanisms of chemotherapy resistance in head and neck cancers. Front Oncol.

[45] Lin X, Li L, Li S, Li Q, Xie D, Zhou M (2021). Targeting the opening of mitochondrial permeability transition pores potentiates nanoparticle drug delivery and mitigates cancer metastasis. Adv Sci.

[46] Czabotar PE, Garcia-Saez AJ (2023). Mechanisms of Bcl-2 family proteins in mitochondrial apoptosis. Nat Rev Mol Cell Biol.

[47] Mullauer FB, Kessler JH, Medema JP (2009). Betulinic acid induces cytochrome c release and apoptosis in a Bax/Bak-independent, permeability transition pore dependent fashion. Apoptosis.

[48] Chernyak BV (1997). Redox regulation of the mitochondrial permeability transition pore. Biosci Rep.

[49] Tafani M, Minchenko DA, Serroni A, Farber JL (2001). Induction of the mitochondrial permeability transition mediates the killing of HeLa cells by staurosporine. Cancer Res.

[50] Ma Q, Xu Y, Tang L, Yang X, Chen Z, Wei Y (2020). Astragalus polysaccharide attenuates cisplatin-induced acute kidney injury by suppressing oxidative damage and mitochondrial dysfunction. Biomed Res Int.

[51] Quarato G, Llambi F, Guy CS, Min J, Actis M, Sun H (2022). Calcium mediated mitochondrial inner membrane permeabilization induces cell death independently of Bax and Bak. Cell Death Differ.

[52] Kroemer G, Martin SJ (2005). Caspase-independent cell death. Nat Med.

[53] Liu X, Fu R, Pan Y, Meza-Sosa KF, Zhang Z, Lieberman J (2018). PNPT1 release from mitochondria during apoptosis triggers decay of Poly(A) RNAs. Cell.

[54] Yu CH, Davidson S, Moghaddas F, Tyebji S, Bye CR, Masters SL (2020). TDP-43 triggers mitochondrial DNA release via mPTP to activate cGAS/STING in ALS. Cell.

[55] Heilig R, Lee J, Tait SWG (2023). Mitochondrial DNA in cell death and inflammation. Biochem SocTrans.

[56] Haider T, Pandey V, Banjare N, Gupta PN, Soni V (2020). Drug resistance in cancer: mechanisms and tackling strategies. Pharm Rep.

[57] Schinzel AC, Takeuchi O, Huang ZH, Fisher JK, Zhou ZP, Rubens J (2005). Cyclophilin D is a component of mitochondrial permeability transition and mediates neuronal cell death after focal cerebral ischemia. Proc Natl Acad Sci USA.

