# Supplementary material for: Nano-flow cytometry unveils mitochondrial permeability transition process and multi-pathway cell death induction for cancer therapy
Source: Cell Death Discov. 2024 Apr 15;10:176. doi: 10.1038/s41420-024-01947-y (PMC11018844; doi:10.1038/s41420-024-01947-y)
Supplement: Supplementary file 1 — Supplementary material [file 41420_2024_1947_MOESM1_ESM.pdf]

# Supplementary Information

# Nano-Flow Cytometry Unveils Mitochondrial Permeability Transition Process and Multi-Pathway Cell Death Induction for Cancer Therapy

Liyun Su[1,2], Jingyi Xu[1,2], Cheng Lu[1], Kaimin Gao[1], Yunyun Hu[1], Chengfeng Xue[1], Xiaomei Yan[1]

[1]Department of Chemical Biology, MOE Key Laboratory of Spectrochemical Analysis & Instrumentation, Key Laboratory for Chemical Biology of Fujian Province, State Key Laboratory of Physical Chemistry of Solid Surfaces, Collaborative Innovation Center of Chemistry for Energy Materials, College of Chemistry and Chemical Engineering, Xiamen University, Xiamen, Fujian 361005, People's Republic of China

[2]L.S. and J.X. contributed equally to this work

To whom correspondence may be addressed. E-mail: xmyan@xmu.edu.cn

**This PDF file includes:**

Expanded Materials and Methods

Fig. S1 to S18

References

**Materials and reagents.** Plasmids of the puromycin resistant lentiviral control shRNA vector (pLKO.1-puro), Bak shRNA vector, and Bax shRNA vector, were purchased from Public Protein/Plasmid Library (PPL, Jiangsu, China). Fetal bovine serum was obtained from Gibco (Grand Island, New York). The mitochondria isolation kit for cultured cells, bicinchoninic acid (BCA) kit, SYTO 62 nucleic acid stain, MitoProbe Transition Pore Assay Kit, and Alexa Fluor 488 Annexin V/Dead cell Apoptosis Kit were purchased from Molecular Probes (Eugene, OR, USA). The following monoclonal antibodies were used: anti-porin monoclonal antibody (mAb), anti-actin, anti-PNPT1, anti-Hsp60, anti-Bak, and anti-Bax were obtained from abcam (Cambridge, MA, USA); anti-Cyt c was obtained from eBiosciences (San Diego, CA, USA); anti-Bcl-2, and anti-Bcl-xL were obtained from Biolegend (San Diego, CA, USA); and anti-AIF mAb was obtained from Beyotime (Haimen, Jiangsu, China). Secondary goat anti-rabbit IgG conjugated to CoraLite 488 antibody was purchased from Proteintech (Wuhan, Hubei, China). Horseradish-peroxidase (HRP)-conjugated secondary antibody was obtained from Multisciences (Hangzhou, China). CDDP and BetA were purchased from Sigma-Aldrich (Saint Louis, Missouri, USA). Staurosporine was purchased from Selleck (Houston, TX, USA). A mitochondrial buffer (MT buffer) containing 250 mM sucrose, 10 mM HEPES, 1 mM EGTA, 4.2 mM sodium succinate hexahydrate, and 1 mM potassium dihydrogen phosphate, and adjusted to pH 7.0 with 1 M potassium hydroxide was used as the negative control and for mitochondria washing and staining. Standard swelling buffer containing 10 mM HEPES, 70 mM sucrose, 210 mM mannitol, 4.2 mM sodium succinate hexahydrate, 1 mM potassium dihydrogen phosphate, and 120 mM KCl adjusted to pH 7.0 with 1 M potassium hydroxide was used to study the mitochondria swelling. Ultrapure water prepared using a Milli-Q RG unit was used in all experiments (Millipore, Bedford, MA, USA). All the other reagents used in present study were of analytical grade and obtained from Sinopharm Chemical Reagent Co., Ltd.

(Shanghai, PRC). All the buffers were filtered through a 0.22-μm filter and used within three weeks.

**Cell Culture.** The HeLa human cervical epitheloid carcinoma cell line, HEK 293T cells, MCF-7, MDA-MB-231 and MCF-7/Dox human breast cancer cell lines were purchased from the American Type Culture Collection (Manassas, VA, USA). HeLa cells, MCF-7, and MCF-7/Dox were cultured in DMEM (Hyclone, Logan, UT, USA) supplemented with 10% fetal bovine serum (FBS) and 1% penicillin/streptomycin (P/S; HyClone) at 37°C in 5% $CO_2$ incubator.

**Isolation of mitochondria.** For each experiment, HeLa cells or HeLa cells with stable expression of shRNA targeting both Bax and Bak (HeLa Bax/Bak shRNA) were cultured and allowed to reach approximately 90% confluency in 15-cm plates. The Cells were then harvested through trypsinization, pelleted, and washed using PBS. To purify the mitochondria, a mitochondria isolation kit designed for cultured cells was employed. The cell pellet was resuspended in 800 μL of cold mitochondria isolation reagent A, and the cells were homogenized with 60 strokes using a dounce tissue grinder on ice. The resulting homogenate was then transferred to a new tube and mixed with 800 μL of cold mitochondria isolation reagent C. To eliminate nuclei and unbroken cells, the homogenate was subjected to centrifugation (700 g, 10 min, 4°C), and the supernatant fraction was repeated one time followed by centrifugation (700 g, 10 min, 4°C). The resulting supernatant fractions were carefully transferred to a new tube and subjected to centrifugation (12 000 g, 15 min, 4°C). This final centrifugation step was carried out to pellet the mitochondrial fraction.

**Determination of mPTP opening.** The opening of mPTP was directly assessed by loading with calcein-AM and $CoCl_2$ in high conductance mode. Briefly, mitochondria were isolated from cultured HeLa cells that had been either treated with a particular drug for a defined duration or left untreated. These mitochondria were then subsequently subjected to an incubation with 5 μM calcein-AM for 30 min at 37°C. Subsequently, the mitochondria were exposed to 400 μM $CoCl_2$ at 37°C for 10 min, centrifuged at 12 000 g for 5 min at 4°C, and washed using mitochondrial buffer (MT buffer). The mitochondria were then resuspended in MT buffer to achieve a final protein concentration of 0.1 mg/mL, before being examined using nFCM analysis.

**Analysis of mitochondrial mtDNA.** For mtDNA staining, following the stimulus, the isolated mitochondria were subjected to centrifugation at 12 000 g for 5 min at 4°C to remove the supernatant. The resulting mitochondrial sample was then pelletized and suspended in 200 μL solution containing 500 nM freshly prepared SYTO 62 working solution in MT buffer. Subsequently, the sample was incubated at 37°C for 30 min and analyzed using nFCM. Quantification and analysis of mtDNA released into the supernatant from mitochondria were performed using a SpectraMax QuickDrop Micro Volume spectrometer (Molecular Devices, San Jose, CA, USA). This method was chosen as nucleic acids predominantly absorb ultraviolet light at a wavelength of 260 nm.

**Analysis of mitochondrial ROS.** The isolated mitochondria were centrifuged at 12 000 g for 5 min at 4°C to remove the supernatant. The resulting mitochondrial pellet was resuspended in 200 μL freshly prepared working solution of MT buffer containing 10 μM MitoSOX Red. After a 30 min incubation at 37°C, the appropriate ROS-inducing stimulant was introduced to initiate the production of ROS. The pre-existing MitoSOX Red in the mixture was used to label the

generated ROS. Subsequent to the stimulus, the isolated mitochondria were centrifuged at 12 000 g for 5 min at 4°C to remove the supernatant. The sample was then resuspended in 50 μL MT buffer and analyzed using nFCM.

**Analysis of mitochondrial membrane potential ($\Delta\Psi_m$).** To assess variations in $\Delta\Psi_m$ resulting from drug treatments, mitochondria were isolated from cells and exposed to distinct stimuli at designed concentrations and time intervals. Subsequently, for $\Delta\Psi_m$ evaluation, the mitochondria were centrifuged at 12 000 g for 5 min to eliminate the drug, and then resuspended in 200 μL of 500 nM $DiOC_6(3)$. This mixture was then subjected to a 30 min incubation at 37°C, washed once with MT buffer, and analyzed by nFCM.

**Analysis of mitochondrial protein.** For immunostaining, the isolated mitochondria after the stimulus were centrifuged and resuspended in 200 μL fixing buffer (1% paraformaldehyde, 250 μg/mL digitonin in MT buffer) at room temperature for 10 min. Subsequently, every 100 μL of the mitochondria suspension was pipetted into an Eppendorf tube and centrifuged. The resulting pellet was resuspended with 50 μL of incubation buffer (250 μg/mL digitonin in MT buffer) containing 20 μg/mL of anti-Cyt c mAb or other specific protein mAb, depending on the experimental requirement. The mitochondrial sample was incubated overnight at 4°C with constant shaking and then washed once with 200 μL of MT buffer. For the second antibody staining, the pellet was reconstituted in 50 μL of incubation buffer containing CoraLite 488-conjugated secondary antibody (1:100 dilution), specific to either mouse or rabbit IgG. After a 2 h incubation at 37°C, the mitochondria were washed twice with 200 μL MT buffer and then resuspended in 50 μL of MT buffer for nFCM analysis. Notably, porin, Bcl-2, and Bcl-xL reside in the OMM with an orientation towards the cytosol, allowing immunofluorescent staining without the need for mitochondrial fixation or permeabilization. However, Cyt c, PNPT1, and

AIF, residing in the IMS, require fixation and permeabilization treatment for antibodies to effectively access intraorganelle proteins.

**Mitochondrial oxygen consumption—Seahorse XFe96 assay.** The oxygen consumption rate (OCR) of isolated mitochondria from HeLa cells was investigated using a Seahorse XFe96 analyzer (Seahorse Biosciences, MA, USA) [1, 2]. The isolated mitochondria were diluted in assay buffer (MAS), composed of mannitol (220 mM), sucrose (70 mM), $KH_2PO_4$ (10 mM), $MgCl_2$ (5 mM), HEPES (2 mM), EGTA (1 mM), and 0.2% BSA (fatty acid free), adjusted to pH 7.2. In the Seahorse 96-well plate, 25 μL of mitochondrial suspension containing 25 μg of protein for both succinate condition and pyruvate/malate condition were added and centrifuged (2000 g × 20 min × 4°C). After centrifugation, 155 μL of MAS containing pyruvate (10 mM) in combination with malate (2 mM) or succinate (10 mM) and rotenone (2 μM) with pH adjusted to 7.2 were added. The plate was immediately analyzed at 37°C. Each condition was performed in six technical replicates for each mitochondrial preparation. For complex I-mediated respiration, glutamate and malate were used as substrates, while complex II-mediated respiration was measured in the presence of rotenone (inhibits complex I) and succinate. Baseline measurements were initially taken before the injection of 4 mM ADP. A 2-minute mixing period was followed by 1 minute of rest, and the measurement lasted 3 minutes. Data were collected and extracted using the Wave software (Agilent Technologies, CA, USA). The protein concentration of the isolated mitochondria was determined using the BCA assay.

**Electron microscopy.** Electron microscopy was employed to observe the differences in mitochondria isolated from HeLa cells across all experimental groups. For each of the three experimental conditions (Control, 400 μM $Ca^{2+}$, 10 μM CsA + 400 μM $Ca^{2+}$), samples of mitochondria were fixed in a solution of 2.5% glutaraldehyde in PBS-TEM (containing 4.37 g

$Na_2HPO_4 \cdot 12H_2O$ and 1.22 g $NaH_2PO_4 \cdot 2H_2O$ dissolved in 200 mL ultrapure water, pH 7.0) for 2 h at 4°C. Subsequently, the fixed mitochondria were washed with PBS-TEM, post-fixed in $OsO_4$ for 1 h at 4°C, dehydrated through an ethanol gradient, infiltrated with EPON, and allowed to polymerize 24–48 h at 60°C. Sections of 50–70 nm were then cut, collected onto 400-mesh high-transmission grids, stained with lead citrate and uranyl acetate, and analyzed using a Tecnai Spirit TEM (FEI, Eindhoven, The Netherlands) operating at 120 kV. The obtained results were processed using Adobe Photoshop software, with linear adjustments applied to brightness and contrast.

**RNA interference and stable cell lines.** The down-regulation of Bax and Bak in HeLa cells was achieved through the utilization of short hairpin RNA (shRNA) interference. The specific target sequences for Bak-specific short hairpin RNA (shRNA) were as follows: Bak-shRNA, 5′-TGGTACGAAGATTCTTCAAAT-3′. Correspondingly, the Bax-specific short hairpin RNA (shRNA) target sequences were: Bak-shRNA, 5′- GACGAACTGGACAGTAACATG-3′. A nontargeted short hairpin RNA (shRNA) with the sequence 5′-CCTAAGGTTAAGTCGCCCTCG-3′ was used as a sham control. Lentivirus particles were harvested 48 h after cotransfection of pLKO.1 Bak shRNA or pLKO.1 Bax shRNA with the packaging plasmid psPAX2 and the VSV-G envelope plasmid pMD2. G (psPAX2 and pMD2. G were purchased from the Public Protein/Plasmid Library, Nanjing, Jiangsu, China) into HEK 293T cells via calcium phosphate transfection. For the silencing experiments, HeLa cells were seeded at a density of $5 \times 10^5$ cells per well in 6-well plates. They were allowed to reach approximately 50% confluence on the day of transfection and were subsequently infected with the resulting recombinant lentivirus in the presence of 10 μg/mL polybrene (Sigma-Aldrich, Saint Louis, Missouri, USA). Two days post-infection, the infected cells were selected using 1 μg/mL puromycin (Sigma-Aldrich, Saint Louis, Missouri, USA). The effectiveness and

specificity of protein knockdown were evaluated through western blotting. Cells transfected with the empty vector served as the control group.

**Western blot analysis.** The cells were collected and lysed in RIPA lysis buffer supplemented with a mixture of protease inhibitors and phosphatase inhibitors (Applygen, Beijing, China) at 4°C for 30 min. The lysates were clarified by centrifugation at 12 000 g for 10 min at 4°C. The protein concentration of the resulting lysates was determined using the BCA assay to ensure equal loading of proteins. Equal amounts of protein (30 μg) were loaded and separated on 12% polyacrylamide gels, and then transferred to PVDF membranes (0.45 μm; Solarbio, Beijing, China). The membranes were blocked using a solution containing 5% nonfat dry milk in TBS for 1 h, followed by incubation with different antibodies overnight at 4°C (cat no. ab14734, 1:1000 mouse monoclonal antibody porin; cat no. ab90529, 1:1000 rabbit polyclonal antibody for Cyt c; cat no. ab32371, 1:3000 rabbit monoclonal antibody for Bak; cat no. ab53154, 1:1000 rabbit polyclonal antibody for Bax; all from Abcam). After the primary antibody incubation, the membrane was washed and then incubated with either HRP-conjugated anti-mouse IgG or anti-rabbit IgG for 1 h at room temperature. Following another wash with TBS, the blots were developed using Meilunbio fg super-sensitive ECL luminescence reagent (Meilunbio, Dalian, Liaoning, China) and imaged using the Amersham Imager 600 (GE Healthcare Life Sciences, Marlborough, MA, USA).

**Assay of cell death.** For the analysis of cell death, Annexin V-FITC/PI staining was performed using an Annexin V-FITC/PI apoptosis detection kit and assessed by flow cytometer (BD FACSAria II, BD Biosciences, Bethedsa, USA) according to the manufacturer's guidelines. Briefly, cells ($1 \times 10^6$ cells/well) were seeded on in 6-well plates. After treatment of cells with the agent, the culture medium was discarded, and the cells were washed with PBS.

Subsequently, the cells were incubated with Annexin V-FITC/PI at 37°C for 15 min in the absence of light. The flow cytometry instrument detected cell fluorescence using a FITC signal detector (FL1) and a PE-Texas Red signal detector (FL2). According to the method, cells that were Annexin V-FITC (-)/PI (-) were considered as survived cells, cells that were Annexin V-FITC (+)/PI (-) were categorized as cells in the early stage of apoptosis, cells that were Annexin V-FITC (+)/PI (+) were identified as cells in the late stage of apoptosis or necrosis, and cells that were Annexin V-FITC (-)/PI (+) were identified as cells in the stage of necrosis. The calculation of cell death percentage includes the sum of three quadrants: Annexin V-FITC (+)/PI (-), Annexin V-FITC (+)/PI (+) and Annexin V-FITC (-)/PI (+). Each experiment was performed in triplicate, and reproducible results were obtained.

**Statistical analysis.** Statistical comparisons are made against the control using Student's t-test. Experiments were performed in triplicate. For all graphs bars represent means ± S.E.M. The significance was taken when P-values were $<0.05$. ****$p < 0.0001$, ***$P < 0.001$, **$P < 0.01$, *$P < 0.05$, and n. s., nonsignificant.

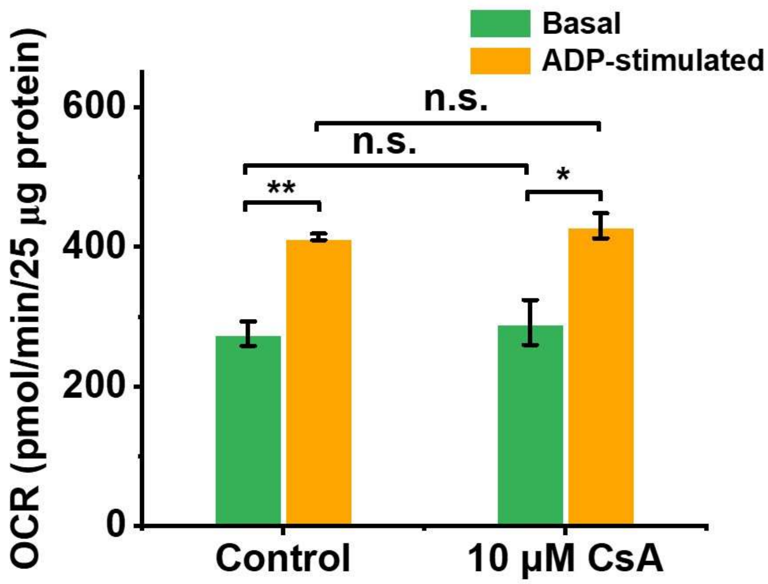


**Supplementary Fig. S1. Assessment of oxidative phosphorylation (OXPHOS) function in isolated mitochondria and the impact of CsA on mitochondrial oxygen consumption rate.** Basal and ADP-stimulated respiration, using substrates for complex I, was measured in isolated mitochondria from HeLa cells using a Seahorse XFe96 analyzer. Measurements were conducted on untreated mitochondria (n = 6) and mitochondria subjected to 10 μM CsA incubation (n = 6). The CsA incubation period was two and a half hours, aligning with the timeframe for effectively inhibiting mPTP opening (30 min of CsA pre-incubation and 2 h of $CaCl_2$ or anti-cancer drug treatment). The ADP concentration used for stimulation was 4 mM, with each test utilizing 25 μg of isolated mitochondria. Statistical significance was determined using paired t-test analysis. **$P < 0.01$, *$P < 0.05$, and n. s., non-significant.

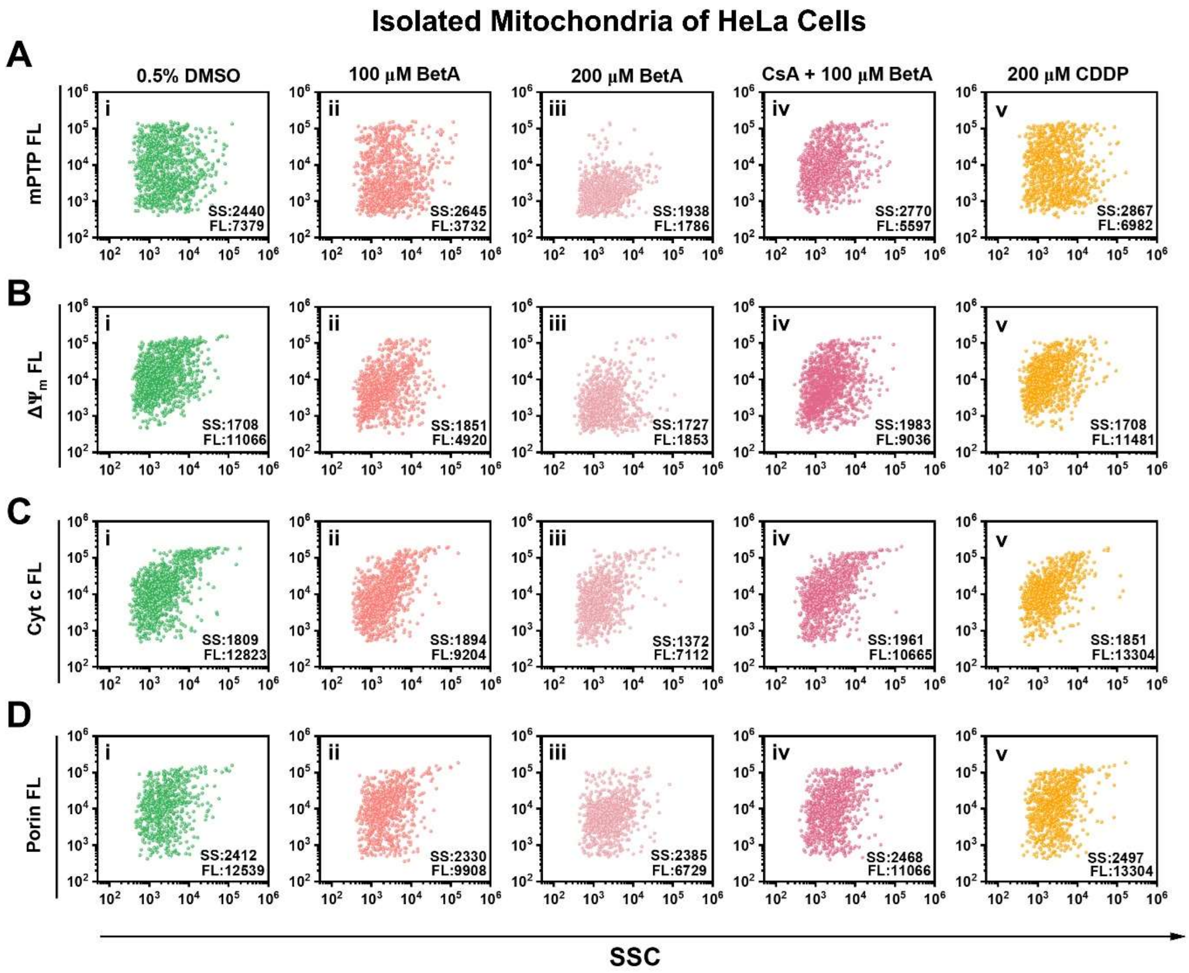


**Supplementary Fig. S2. Identification of direct induction of mPT-mediated dysfunction in isolated mitochondria from HeLa cells by anticancer drugs using nFCM. A-D** Bivariate dot-plots of fluorescence signals for mPTP (**A**), $\Delta\Psi_m$ (**B**), Cyt c (**C**), and porin (**D**) against the side scatter of mitochondria obtained through nFCM. The isolated mitochondria from HeLa cells were treated with 0.5% DMSO (control) (i), 100 μM BetA (ii), 200 μM BetA (iii), 100 μM BetA after pre-incubation with 10 μM CsA for 30 min (iv), and 200 μM CDDP (vi) for 2 h.

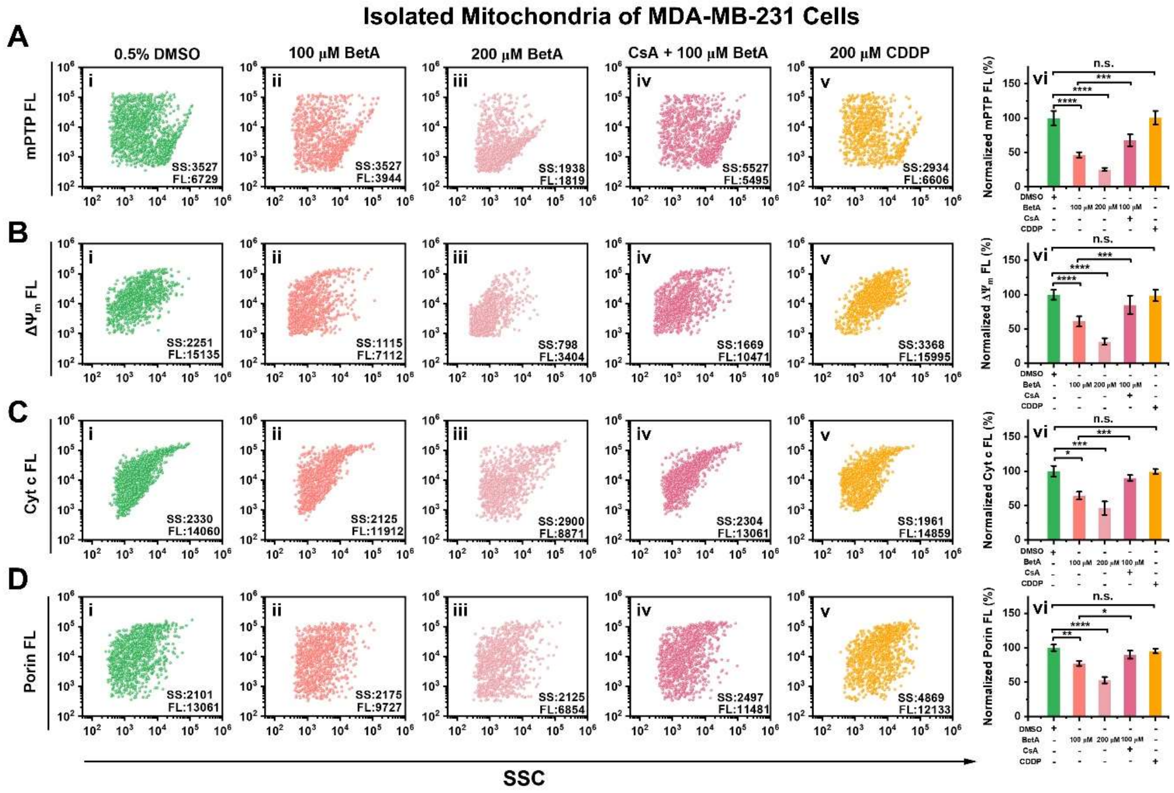


**Supplementary Fig. S3. Identification of direct induction of mPT-mediated dysfunction in isolated mitochondria from MDA-MB-231 cells by anticancer drugs using nFCM. A-D** Bivariate dot-plots of fluorescence signals for mPTP (**A**), $\Delta\Psi_m$ (**B**), Cyt c (**C**), and porin (**D**) against the side scattering of mitochondria obtained through nFCM. Isolated mitochondria from MDA-MB-231 cells were treated with 0.5% DMSO (control) (i), 100 μM BetA (ii), 200 μM BetA (iii), 100 μM BetA after pre-incubation with 10 μM CsA for 30 min (iv), and 200 μM CDDP (v) for 2 h. The corresponding normalized bar graphs are presented (vi) for each analyzed parameter. Error bars represent the mean ± standard error from three independent experiments, and statistical significance was determined using paired t-test analysis. ****p < 0.0001, ***P < 0.001, **P < 0.01, *P < 0.05, and n. s., non-significant.

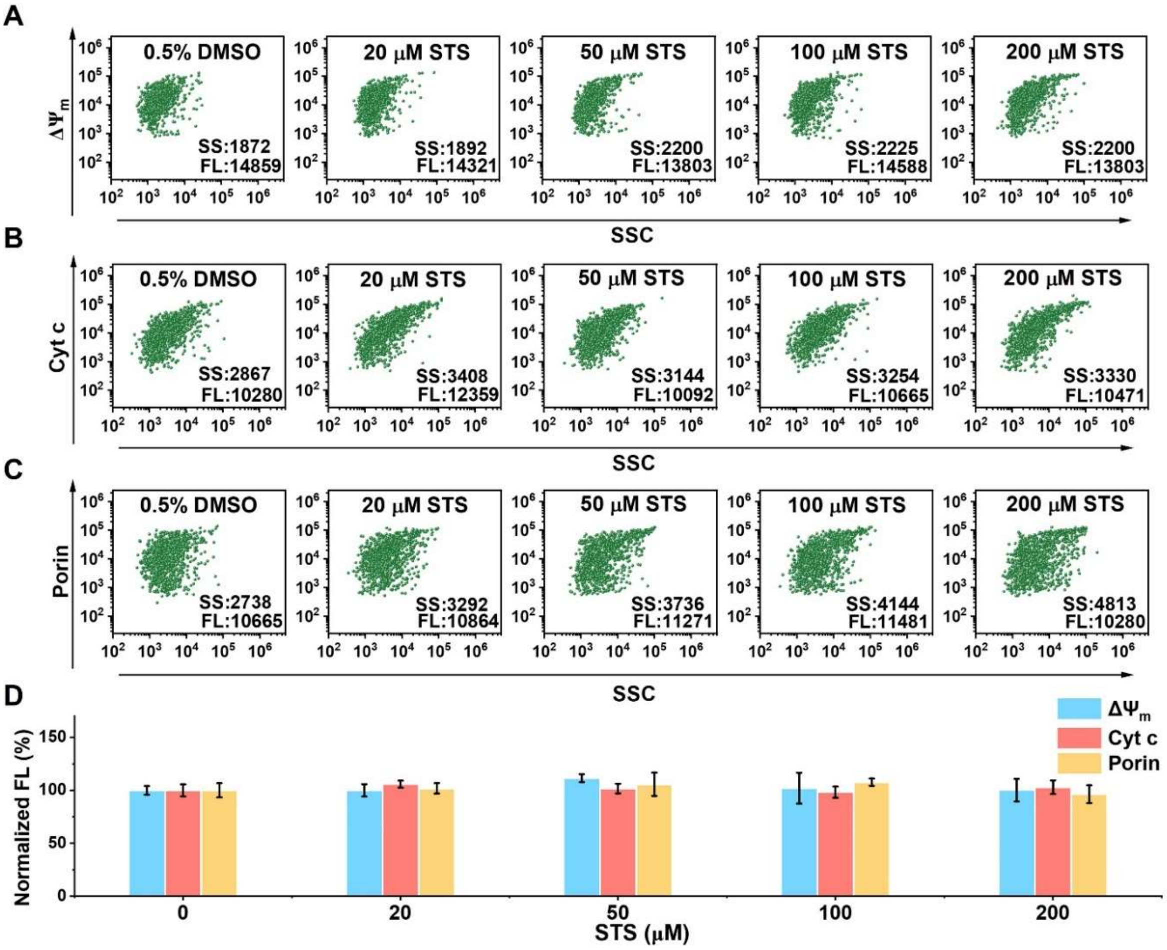


**Supplementary Fig. S4. Analysis of the staurosporine (STS) concentration effect on isolated mitochondria by nFCM. A-C** The bivariate dot-plots of the $\Delta\Psi_m$ (**A**), Cyt c (**B**), and porin (**C**) green fluorescence burst area versus the SSC burst area for isolated mitochondria treated with 0, 20, 50,100, and 200 μM STS for 2 h. **D** Normalized fluorescence burst area of $\Delta\Psi_m$, Cyt c, and porin for isolated mitochondria treated with different concentrations of STS.

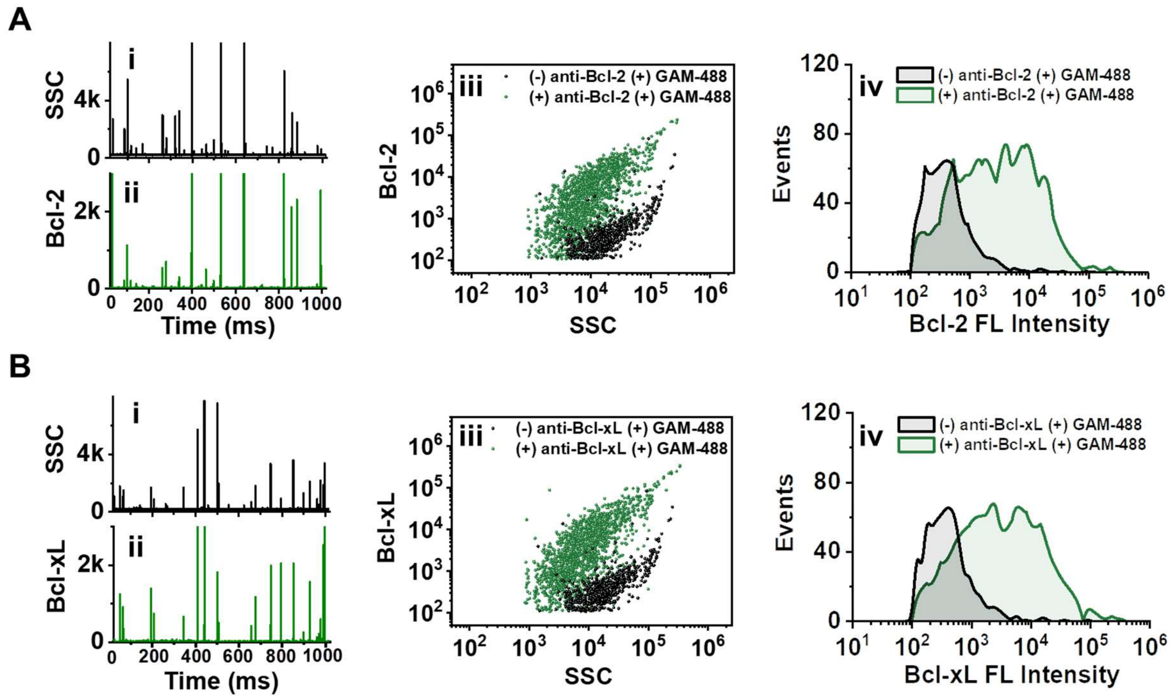


**Supplementary Fig. S5. nFCM analysis of mitochondria stained with or without anti-Bcl-2 mAb and anti-Bcl-xL mAb, followed by CoraLite 488-conjugated GAM labeling. A** Representative burst traces of side scatter (i) and Bcl-2 fluorescence (ii), bivariate dot-plot of Bcl-2 burst area versus side scatter burst area (iii), and histograms depicting side scatter intensity and Bcl-2 fluorescence distribution (iv) of isolated mitochondria. **B** Representative burst traces of side scatter (i) and Bcl-xL fluorescence (ii), bivariate dot-plot of Bcl-xL burst area versus side scatter burst area (iii), and histograms depicting side scatter intensity and Bcl-xL fluorescence distribution (iv) of isolated mitochondria.

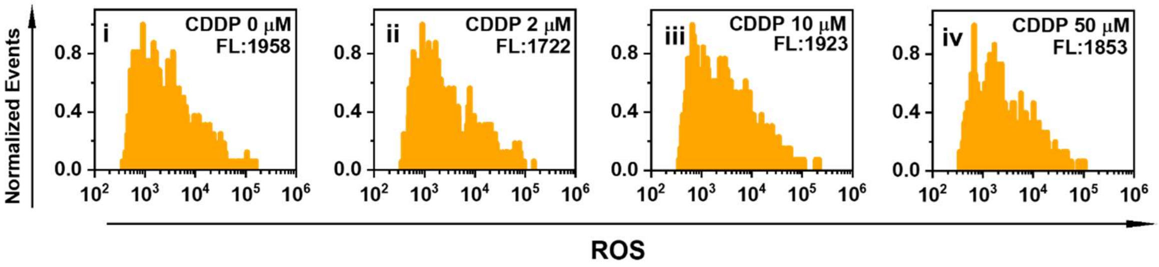


**Supplementary Fig. S6. nFCM analysis of mitochondrial ROS production following CDDP treatment.** Histograms depicting ROS fluorescence distribution of isolated mitochondria treated with 0, 2, 10, and 50 μM CDDP for 120 min.

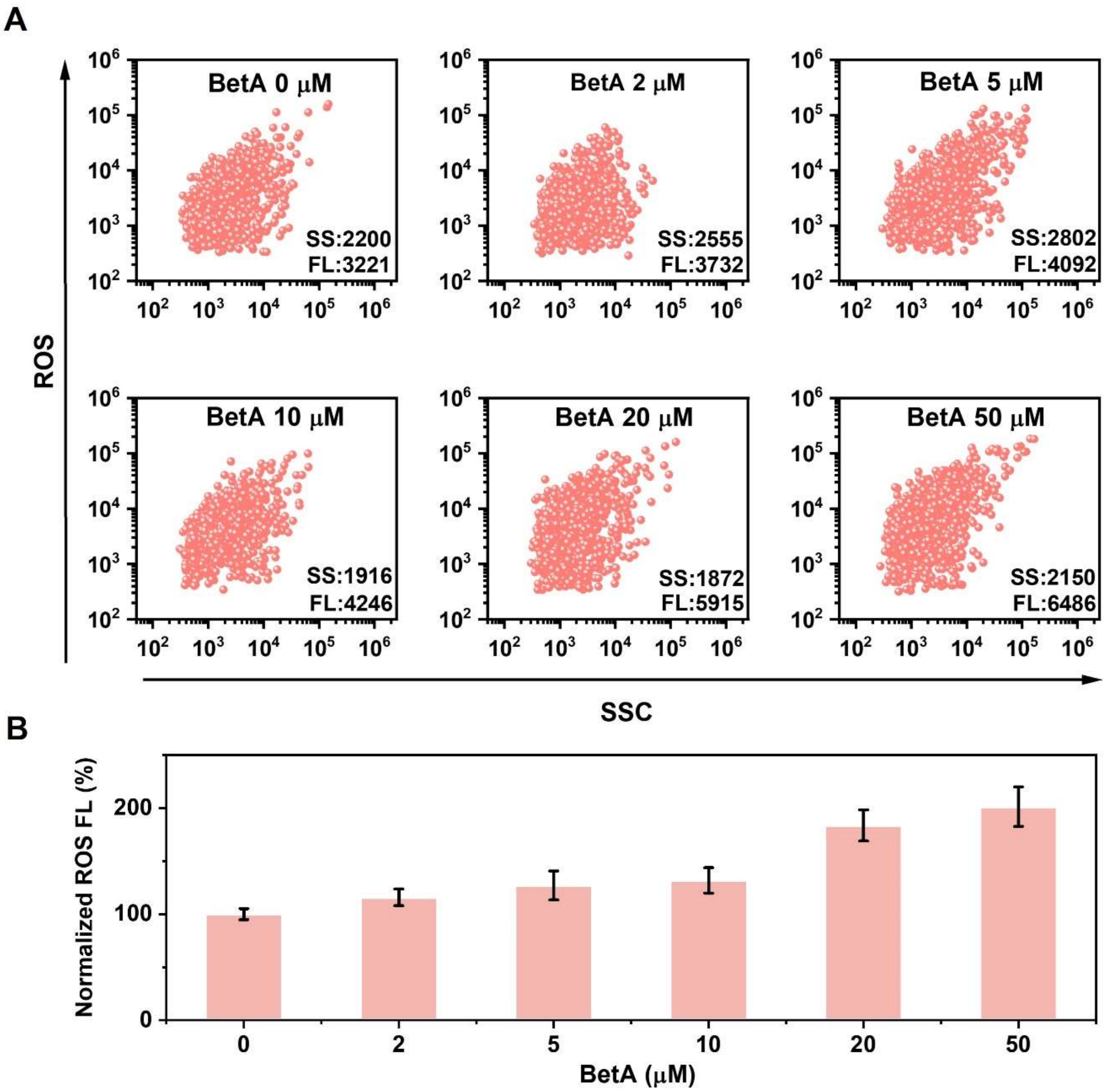


**Supplementary Fig. S7. nFCM analysis of mitochondrial ROS production following 5 min of BetA stimulation. A** Bivariate dot-plots of the ROS red fluorescence burst area versus the side scatter burst area for isolated mitochondria exposed to 0, 2, 5, 10, 20, and 50 μM BetA for 5 min. **B** Influence of drug concentration on ROS levels within isolated mitochondria upon treatment with BetA.

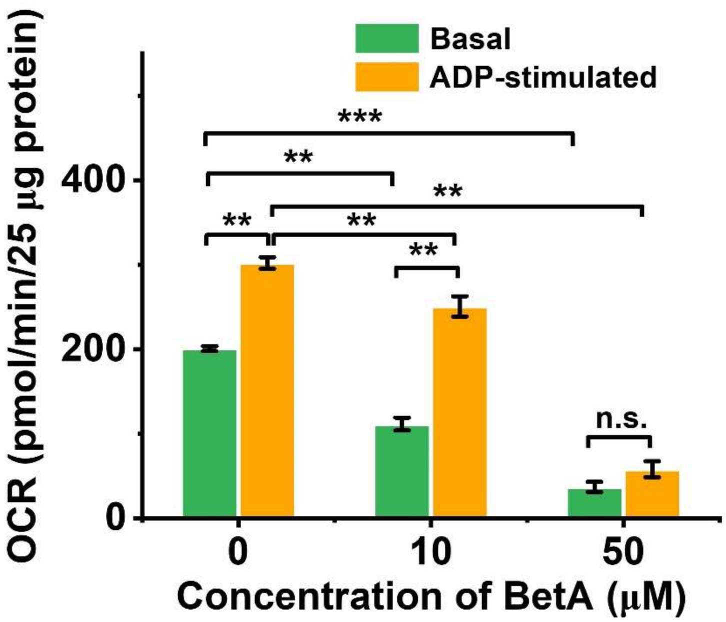


**Supplementary Fig. S8. Assessment of BetA impact on mitochondrial oxygen consumption rate.** Basal and ADP-stimulated respiration, employing substrates for complex II, was quantified in isolated mitochondria from HeLa cells using a Seahorse XFe96 analyzer. Measurements were conducted on untreated mitochondria ($n = 6$) and mitochondria treated with 10 μM ($n = 6$) and 50 μM BetA ($n = 6$) after a 2-h incubation period. Stimulation was achieved with 4 mM ADP, and each assay utilized 25 μg of isolated mitochondria. Paired t-test analysis determined statistical significance. $^{**}P < 0.01$, $^{*}P < 0.05$, and n. s., non-significant.

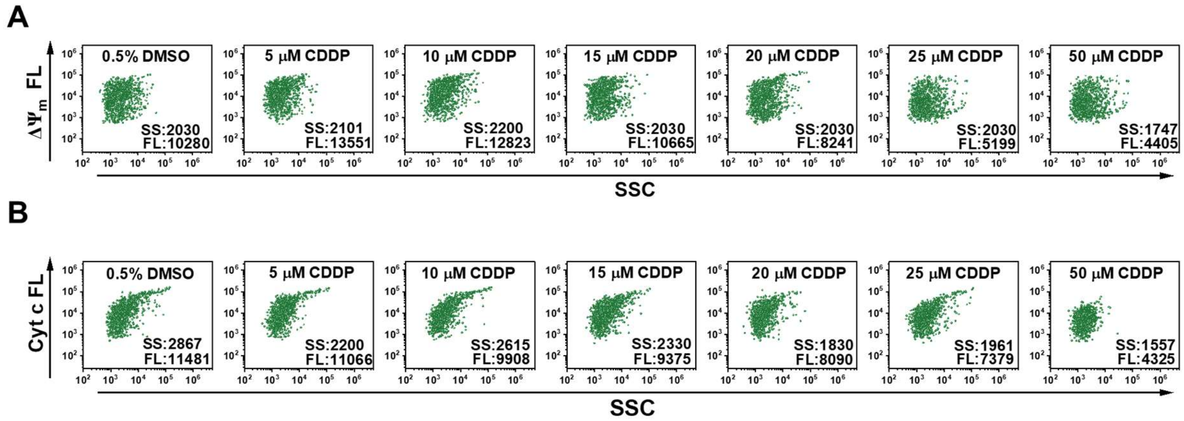


**Supplementary Fig. S9. nFCM analysis of mitochondrial $\Delta\Psi_m$ loss and Cyt c release treated with different concentrations of CDDP on HeLa cells. A** Bivariate dot-plots of the $\Delta\Psi_m$ green fluorescence burst area versus the side scatter burst area for mitochondria isolated from HeLa cells that has been treated with 0, 5, 10, 15, 20, 25, and 50 μM CDDP for 24 h. **B** The bivariate dot-plots of the Cyt c green fluorescence burst area versus the side scatter burst area for mitochondria isolated from HeLa cells that has been treated with 0, 5, 10, 15, 20, 25, and 50 μM CDDP for 24 h.

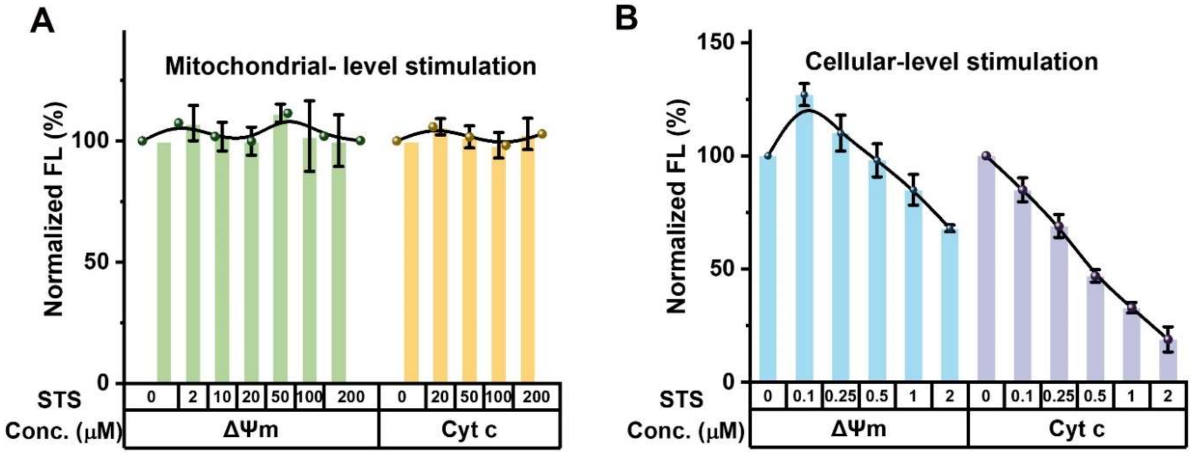


**Supplementary Fig. S10. The sequence of $\Delta\Psi_m$ collapse and Cyt c release upon stimulation with different concentrations of STS at the mitochondrial or cellular level.** **A** Isolated mitochondria were stimulated by different concentrations of STS for 2 h, and the changes in $\Delta\Psi_m$ and Cyt c were analyzed at the single-mitochondrion level using nFCM. **B** The mitochondria of HeLa cells were purified after stimulation with different concentrations of STS at the cellular level for 24 h, and the changes in $\Delta\Psi_m$ and Cyt c were analyzed at the single-mitochondrion level using nFCM.

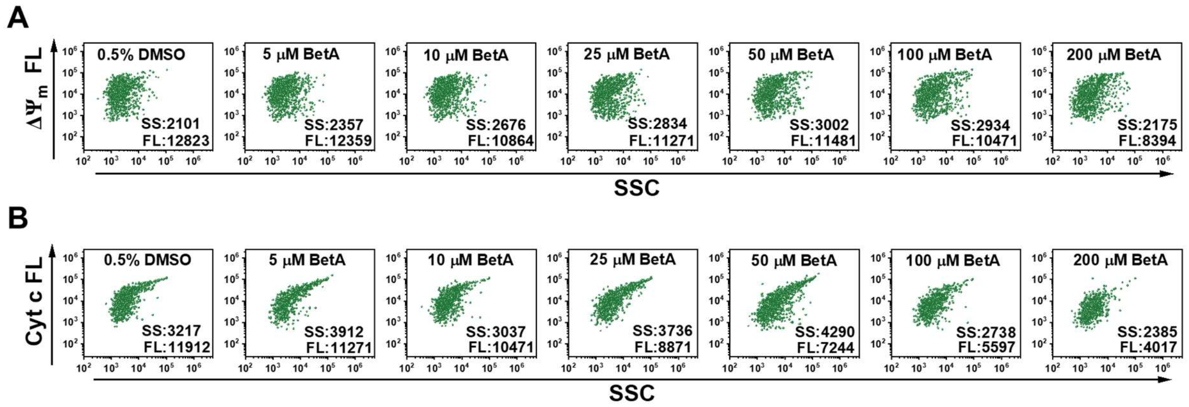


**Supplementary Fig. S11. nFCM analysis of mitochondrial $\Delta\Psi_m$ loss and Cyt c release in mitochondria isolated from HeLa cells treated with different concentrations of BetA. A** Bivariate dot-plots of the $\Delta\Psi_m$ green fluorescence burst area versus the side scatter burst area for mitochondria isolated from HeLa cells treated with 0, 5, 10, 25, 50, 100 μM, and 200 μM BetA for 24 h. **B** Bivariate dot-plots of the Cyt c green fluorescence burst area versus the side scatter burst area for mitochondria isolated from HeLa cells treated with 0, 5, 10, 25, 50, 100 μM, and 200 μM BetA for 24 h.

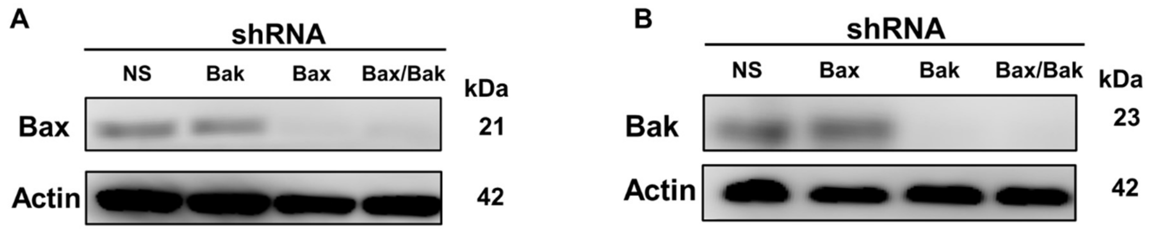


**Supplementary Fig. S12.** Western blot analysis of the Bax (**A**) and Bak (**B**) protein contents in Bax shRNA, Bak shRNA, and Bax/Bak shRNA HeLa cells.

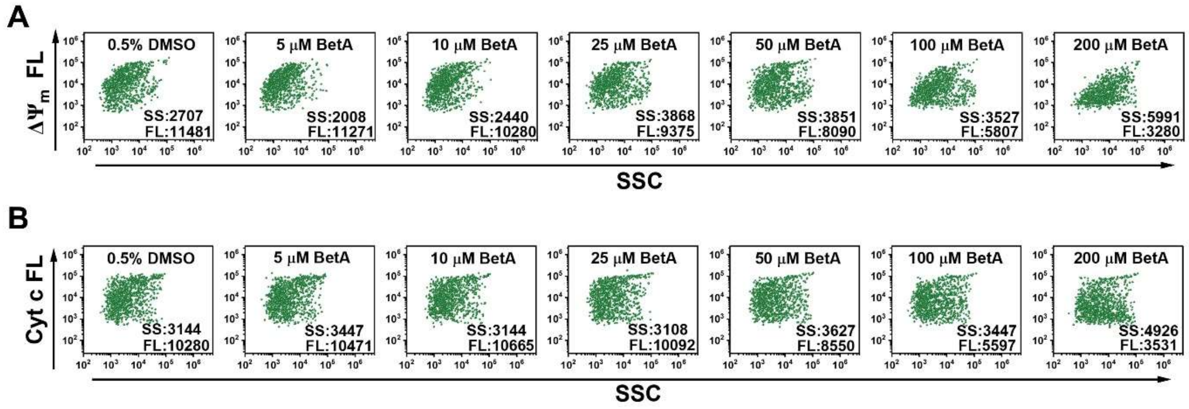


**Supplementary Fig. S13. nFCM analysis of mitochondrial $\Delta\Psi_m$ loss and Cyt c release in mitochondria isolated from Bax/Bak shRNA HeLa cells treated with different concentrations of BetA. A** Bivariate dot-plots of the $\Delta\Psi_m$ green fluorescence burst area versus the side scatter burst area for mitochondria isolated from Bax/Bak shRNA HeLa cells treated with 0, 5, 10, 25, 50, 100 μM, and 200 μM BetA for 24 h. **B** Bivariate dot-plots of the Cyt c green fluorescence burst area versus the side scatter burst area for mitochondria isolated from Bax/Bak shRNA HeLa cell treated with 0, 5, 10, 25, 50, 100 μM, and 200 μM BetA for 24 h.

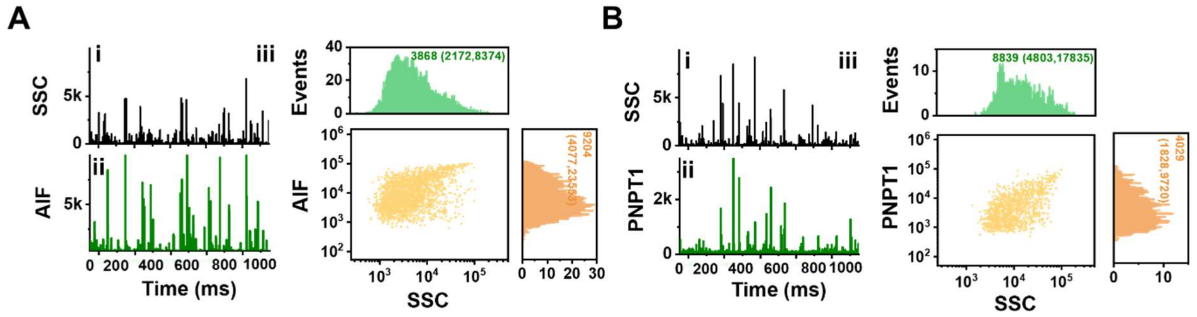


**Supplementary Fig. S14. nFCM analysis of AIF and PNPT1 at the single-mitochondrion level. A** Representative burst traces of side scatter (i) and AIF green fluorescence (ii), along with bivariate dot-plot of the AIF green fluorescence burst area versus the side scatter burst area (iii) for mitochondria isolated from HeLa cells. **B** Representative burst traces of side scatter (i) and PNPT1 green fluorescence (ii), along with bivariate dot-plot of the PNPT1 green fluorescence burst area versus the side scatter burst area (iii) for mitochondria isolated from HeLa cells.

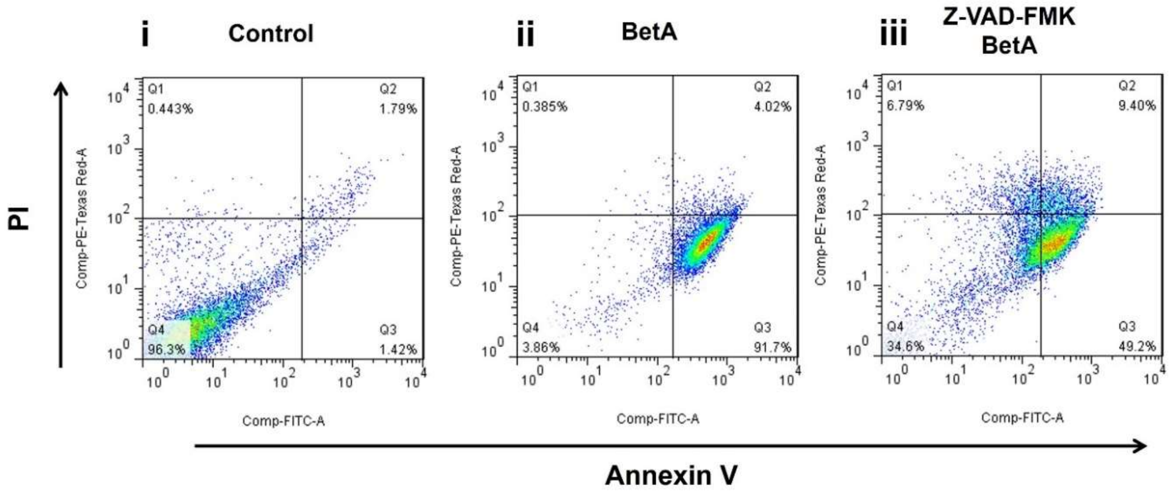


**Supplementary Fig. S15. BetA induces caspase-independent cell death.** Cells without drug stimulation served as the control group (i). HeLa cells were pre-treated without (ii) or with (iii) 100 μM Z-VAD (OMe)-FMK for 12 h before BetA (100 μM) addition. After 24 h, cell death was then assessed by quantifying Annexin V/PI staining using flow cytometric analysis.

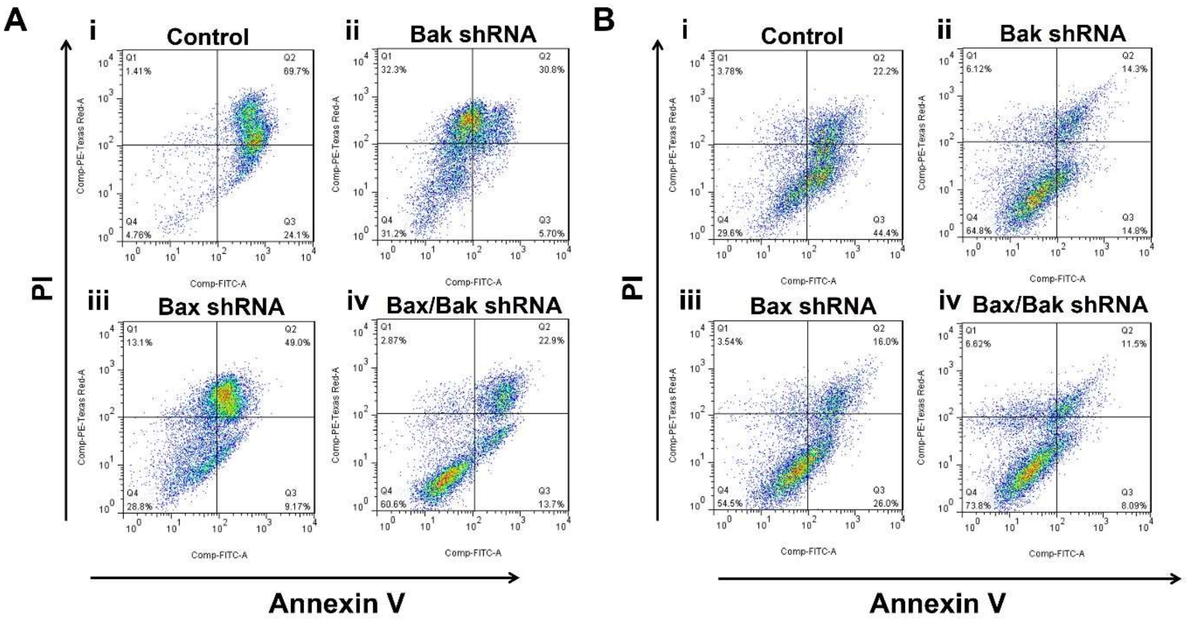


**Supplementary Fig. S16. Analysis of cell death induced by different drugs in various kinds of cells using the BD FACSAria flow cytometer.** HeLa cells infected with lentivirus carrying a scrambled shRNA (control, i) or shRNAs of human Bak (Bak shRNA, ii), Bax (Bax shRNA, iii), or both (Bax/Bak shRNA, iv) were treated with 100 μM CDDP (**A**) or 500 nM STS (**B**) for 24 h. Cell death was then assessed by quantifying Annexin V/PI staining using flow cytometric analysis.

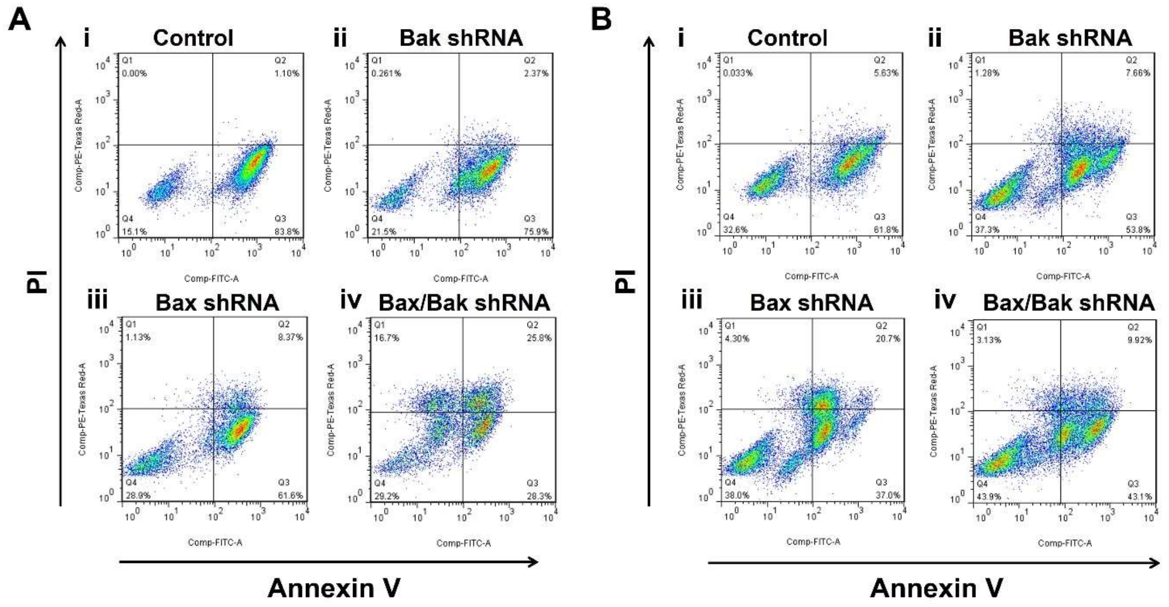


**Supplementary Fig. S17. Analysis of cell death induced by different drugs in various kinds of cells using the BD FACSAria flow cytometer.** HeLa cells infected with lentivirus carrying a scrambled shRNA (control, i) or shRNAs of human Bak (Bak shRNA, ii), Bax (Bax shRNA, iii), or both (Bax/Bak shRNA, iv) were treated with 100 μM BetA (**A**) or 100 μM AA (**B**) for 24 h. Cell death was then assessed by quantifying Annexin V/PI staining using flow cytometric analysis.

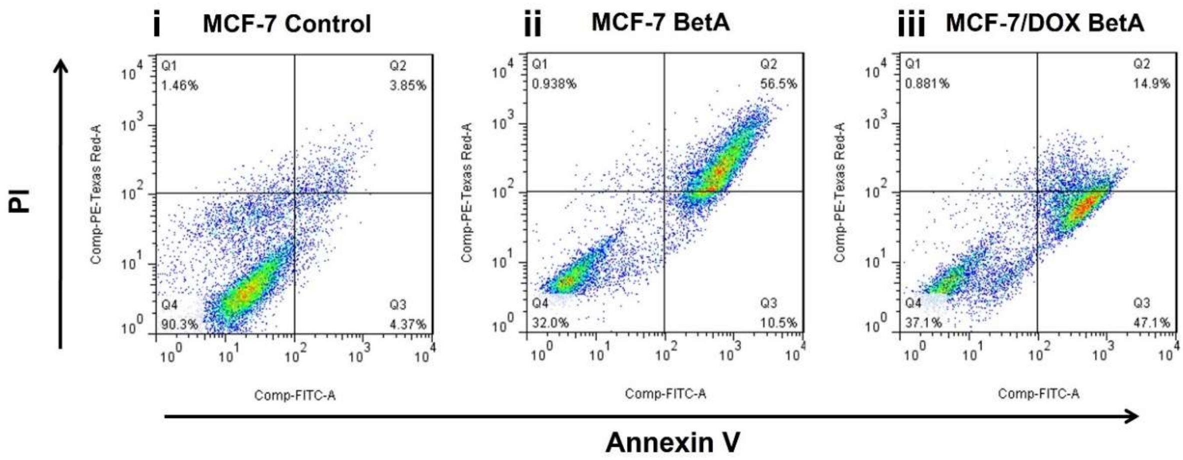


**Supplementary Fig. S18. Analysis of cell death induced by BetA in different cancer cell lines using the BD FACSAria flow cytometer.** MCF-7 cells without drug stimulation served as the control group (i). MCF-7 (ii) and MCF-7 DOX cells (iii) were treated with 100 μM BetA for 24 h. Cell death was then assessed by quantifying Annexin V/PI staining using flow cytometric analysis.

## References:

1. Andersen JV, Jakobsen E, Waagepetersen HS, Aldana BI. Distinct differences in rates of oxygen consumption and ATP synthesis of regionally isolated non-synaptic mouse brain mitochondria. J Neurosci Res. 2019;97:961-974.

2. Iuso A, Repp B, Biagosch C, Terrile C, Prokisch H. Assessing Mitochondrial Bioenergetics in Isolated Mitochondria from Various Mouse Tissues Using Seahorse XF96 Analyzer. Methods Mol Biol. 2017;1567:217-230.
