# Supplementary material for: Nano-flow cytometry unveils mitochondrial permeability transition process and multi-pathway cell death induction for cancer therapy
Source: Cell Death Discov. 2024 Apr 15;10:176. doi: 10.1038/s41420-024-01947-y (PMC11018844; doi:10.1038/s41420-024-01947-y)

Unprocessed western blots for indicated figures panels.

Figure 1C

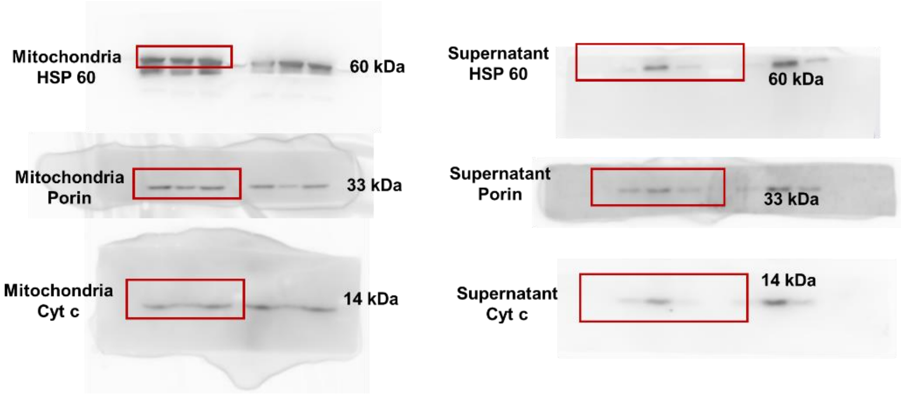

Figure 4C i and ii

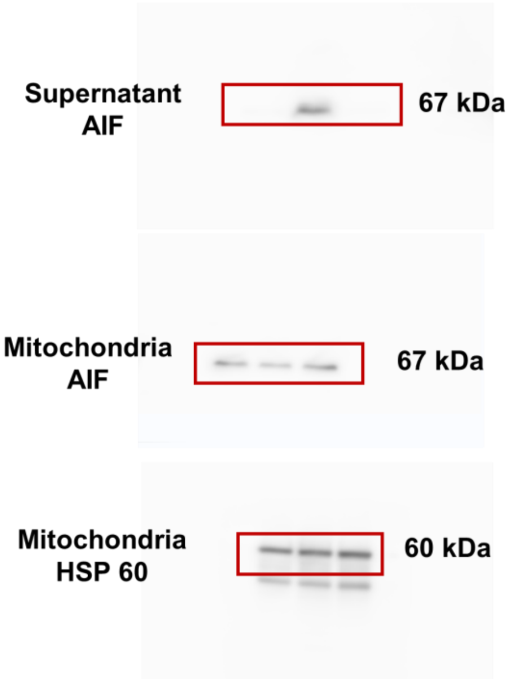

Figure 4C ii

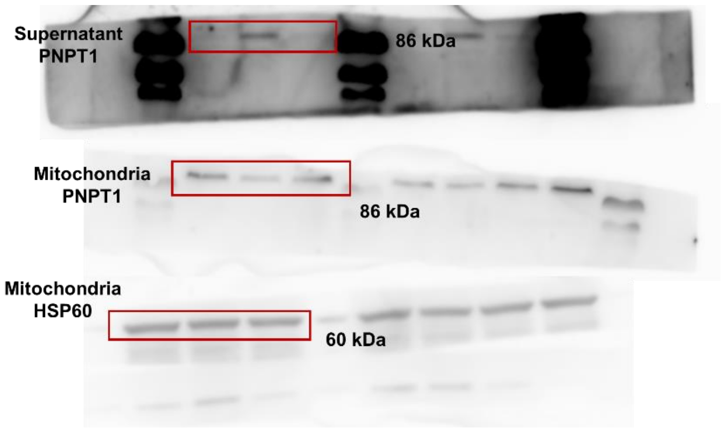

Supplementary Fig. S12 A, B

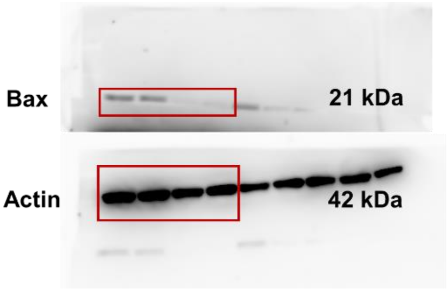

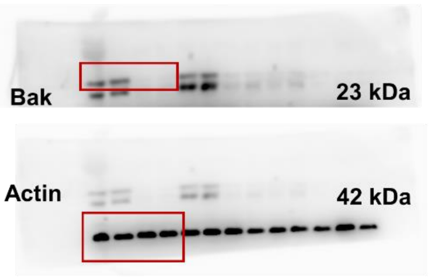

Supplement: Supplementary file 2 — WB Original file [file 41420_2024_1947_MOESM2_ESM.pdf]
